# Adsorption-dominated interfacial regulation by sarcosine via hydrogen-bond water modulation for stable Zn metal anodes

**DOI:** 10.3389/fchem.2026.1986660

**Published:** 2026-10-02

**Authors:** Zijing Wang, Haolin Ju, Hao Zhang, Yiqian Yang, Huan Liu, Bin Cao, Jie Ding, Bin Xu

**Affiliations:** 1 College of Materials Science and Engineering, Xi’an University of Science and Technology, Xi’an, China; 2 State Key Laboratory of Organic Inorganic Composites, Beijing Key Laboratory of Electrochemical Process and Technology for Materials, Beijing University of Chemical Technology, Beijing, China; 3 Institute of Advanced Energy Storage Materials and Technologies, School of Chemistry and Chemical Engineering, Yan’an University, Yan’an, China

**Keywords:** adsorption, aqueous zinc-ion batteries, hydrogen-bond water regulation, sarcosine, zn metal anode

## Abstract

Aqueous zinc-ion batteries (AZIBs) hold great promise for safe and low‐cost energy storage, yet their practical application is seriously hindered by the hydrogen evolution reaction (HER), uncontrolled dendrite growth, and inert byproduct (Zn4SO4(OH)6·xH2O) deposition on the Zn anode. In this work, sarcosine (Sar) was adopted as a multifunctional bipolar electrolyte additive, and first‐principles calculations combined with electrochemical tests and interfacial characterizations were performed to reveal its adsorption‐dominated interfacial stabilization mechanism. Sar strongly adsorbs on the Zn surface to homogenize Zn2+ flux, regulate the electric field distribution, and reconfigure the interfacial environment. Meanwhile, Sar forms strong intermolecular hydrogen bonds with water molecules via its amino and carboxyl groups, reducing free water activity and suppressing HER. First‐principles calculations verify the favorable adsorption configuration and high adsorption energy of Sar on the Zn surface. As a result, the Zn||Zn symmetric cell delivers ultra‐long cycling stability over 2100 h at 1 mA cm‐2, and the Zn||Cu half‐cell exhibits a high average Coulombic efficiency of 99.7%. The full cell with ZnI@CNT‐AC cathode maintains excellent capacity retention after 3000 cycles. This work provides a simple, green, and low‐cost strategy for stabilizing Zn anodes toward practical aqueous zinc‐ion batteries.

## 1 Introduction

Aqueous zinc-ion batteries (AZIBs) are regarded as highly promising electrochemical energy-storage devices for large-scale energy storage owing to their merits including Zn-metal anode with high theoretical capacity, low cost, intrinsic safety, and environmental benignity (Jiang Q. et al., 2025; Li et al., 2025d ; Liu X. et al., 2026; Liu et al., 2026; Lv et al., 2026; Ma et al., 2024; Xu et al., 2025). These advantages enable AZIBs to stand out among various emerging aqueous energy-storage technologies. However, the thermodynamic and kinetic instability of Zn-metal anodes in aqueous electrolytes induces severe interfacial side reactions and dendrite growth, which greatly restrict the practical commercialization of AZIBs (Cai et al., 2026; Jin et al., 2026; Yi et al., 2026; Yuan et al., 2026). First, inevitable reduction of water molecules during Zn electrodeposition triggers vigorous hydrogen evolution reaction (HER). This is because the hydrogen evolution potential at pH = 5 (−0.296 V vs. standard hydrogen electrode) is more positive than the redox potential of Zn^2+^/Zn (−0.76 V vs. standard hydrogen electrode) (Ai et al., 2026; Xia et al., 2026). This process not only causes electrolyte depletion and battery swelling, but also accelerates corrosion of the Zn-metal anode and reduces Zn utilization. Second, uneven distribution of the interfacial electric field leads to continuous dendrite growth during repeated Zn plating/stripping (Guo et al., 2025; Li M. et al., 2026; Zhao M.‐J. et al., 2026; Zhou et al., 2026). In addition, hydroxide ions generated by hydrolysis promote the formation of insoluble and electrochemically inactive byproducts (e.g., ZnSO_4_(OH)_6_·xH_2_O) (Zhang et al., 2026b; Zhou et al., 2026), resulting in heterogeneous electrode surface morphology, excessive consumption of Zn^2+^, and further aggravated dendrite formation (Lim et al., 2026; Zhang et al., 2026a; Zhuang et al., 2026). Interfacial issues such as HER, dendrite growth, and byproduct accumulation are mainly responsible for the low Coulombic efficiency, short cycle life, and poor Zn utilization of AZIBs. Therefore, constructing a stable Zn anode/electrolyte interface and deeply understanding interfacial mass transport and charge-transfer processes are urgently imperative for the practical application of AZIBs. Nevertheless, tackling these interfacial issues remains tremendously challenging.

Although extensive modification strategies have been developed worldwide to address the interfacial instability of Zn-metal anodes, critical bottlenecks remain in regulating the anode–electrolyte interfacial chemistry of AZIBs. Representative approaches, including artificial protective coating (Chang et al., 2025; Du et al., 2026; Liu Z. L. et al., 2026; Peng M. et al., 2026; Yang et al., 2025), concentrated electrolyte design (Liu H. et al., 2026; Qu et al., 2023; Zhao S. et al., 2026) and conventional electrolyte additives (Chen et al., 2025; Hu et al., 2025; Jiang L. et al., 2025; Li et al., 2025b; Li T. et al., 2026; Liang et al., 2026; Sun et al., 2026; Xue et al., 2025), all suffer from inherent limitations in cycling durability, manufacturing cost, or solvation modulation efficacy (Chang et al., 2025; Li et al., 2025a; Li et al., 2025c).To this end, there is an urgent demand for low-cost, high-efficiency additive candidates capable of achieving robust interfacial adhesion and stable functional interphase construction. Natural amino acids and their derivatives have attracted growing research attention as electrolyte additives for AZIBs in recent years (Lu et al., 2021; Peng Z. et al., 2026; Xiong et al., 2025; Zhang et al., 2025). Enriched with polar functional groups such as amino and carboxyl moieties, these molecules can form multiple interactions with Zn^2+^ and H_2_O, thereby tailoring the interfacial behavior of Zn-metal anodes (Cao et al., 2024; Liu Z. et al., 2026; Sun et al., 2024; Wu et al., 2024). Compared with previously reported organic additives, which often require complicated synthesis, high cost, or the use of organic solvents, sarcosine is a naturally occurring, inexpensive, water-soluble, and biodegradable amino acid. It stabilizes the Zn anode through a strong adsorption-dominated mechanism without introducing any toxic or corrosive components, offering a more economical and environmentally friendly route for practical aqueous zinc-ion batteries. Moreover, Sar was selected because its compact molecular structure combines amino and carboxyl functionalities, offering interaction sites for both hydrogen bonding with water and adsorption at the Zn/electrolyte interface (Cernei et al., 2013; Zhang et al., 2009). This combination provides a molecular basis for simultaneously regulating the local water environment and Zn deposition, thereby targeting water-induced side reactions and interfacial deposition heterogeneity (Dong et al., 2026; Ni et al., 2026; Yang et al., 2026). Its aqueous solubility further facilitates its incorporation into ZnSO_4_ electrolytes. These considerations motivated us to investigate Sar as an additive for coupling interfacial adsorption with hydrogen-bond regulation to stabilize Zn-metal anodes.

Herein, we rationally design a sarcosine-functionalized electrolyte system to resolve the persistent Zn anode instability arising from the weak interfacial adhesion of conventional additives and the poor mechanical stability of as-formed protective layers. Sar acts as a multifunctional additive that synergistically optimizes the Zn anode interfacial microenvironment via its amino and carboxyl dual groups. On one hand, Sar molecules form intermolecular hydrogen bonds with water molecules to disrupt the intrinsic hydrogen-bond network of bulk water, deactivate highly active free water, and suppress water hydrolysis and parasitic hydrogen evolution reactions. On the other hand, Sar preferentially adsorbs on the Zn surface and weakly interacts with Zn^2+^ in the electrolyte, thereby regulating the local coordination environment and interfacial electric-field distribution without substantially altering the primary Zn^2+^ solvation sheath. Sar is a hydrogen-bond regulating and preferential surface adsorber that reorganizes the Zn/electrolyte interfacial microenvironment and helps to form a protective interphase. Sar induces interfacial restructuring upon oriented adsorption and forms a functional electrode-electrolyte interphase *in situ*. First-principles calculations confirm a strong adsorption energy of Sar on the Zn surface, corroborating the adsorption-dominated mechanism. Through the above synergistic adsorption-mediated interfacial regulation, the sarcosine-modified electrolyte enables enhanced electrochemical performance of AZIBs. The Zn||Zn symmetric cell has a stable service life of 2,100 h, and the Zn||Cu half-cell has an average Coulombic efficiency of 99.7%. The assembled full-cell also shows only a small capacity fade after 3,000 cycles. Sarcosine also reduces the production of electrochemically inactive by-products on the Zn anode and enhances Zn utilization. Based on the above results, a simple, low-cost, and environmentally friendly adsorption-mediated interface regulation strategy has been proposed to stabilize Zn-metal anodes and promote the application of AZIBs.

## 2 Results and discussion

Density Functional Theory calculations were carried out to investigate how Sar modifies the Zn/electrolyte interface in aqueous ZnSO_4_. The map of the molecular electrostatic potential showed a strong polarization with spatially separated positive and negative regions (Figure 1a). The local positive maxima near the N–H and O–H hydrogens were 40.57 and 46.21 kcal mol^-1^, respectively. The O-H region had the most positive potential and thus a strong hydrogen-bond-donating ability. Therefore, the positively polarized O‒H and N‒H sites can bind H_2_O via hydrogen bonding and restrict the mobility of free water. Thus, the water-decomposition and other parasitic reactions in the bulk electrolyte can be reduced. The carbonyl oxygen had a negative electrostatic potential of −38.97 kcal mol^-1^ and was thus electron-rich, containing a lone pair. This electron-rich site provides a plausible anchor for Zn^2+^ coordination and Sar adsorption at the Zn anode. Such interfacial bonding may regulate Zn^2+^ deposition and homogenize the local electric field to promote uniform Zn nucleation and growth. In contrast, the electrostatic potential distribution of H_2_O shows a strong dipolar character with a positive potential around the H atoms and a negative potential around the O atom (Figure 1b), confirming its high interfacial reactivity and strong tendency to participate in side reactions in aqueous electrolytes. Overall, the combined ESP and Frontier orbital analyses demonstrate that Sar can synergistically regulate water activity and Zn interfacial chemistry through strong molecular polarity, hydrogen-bond interactions, preferential interfacial adsorption, and favorable electron-accepting ability.

**FIGURE 1:**
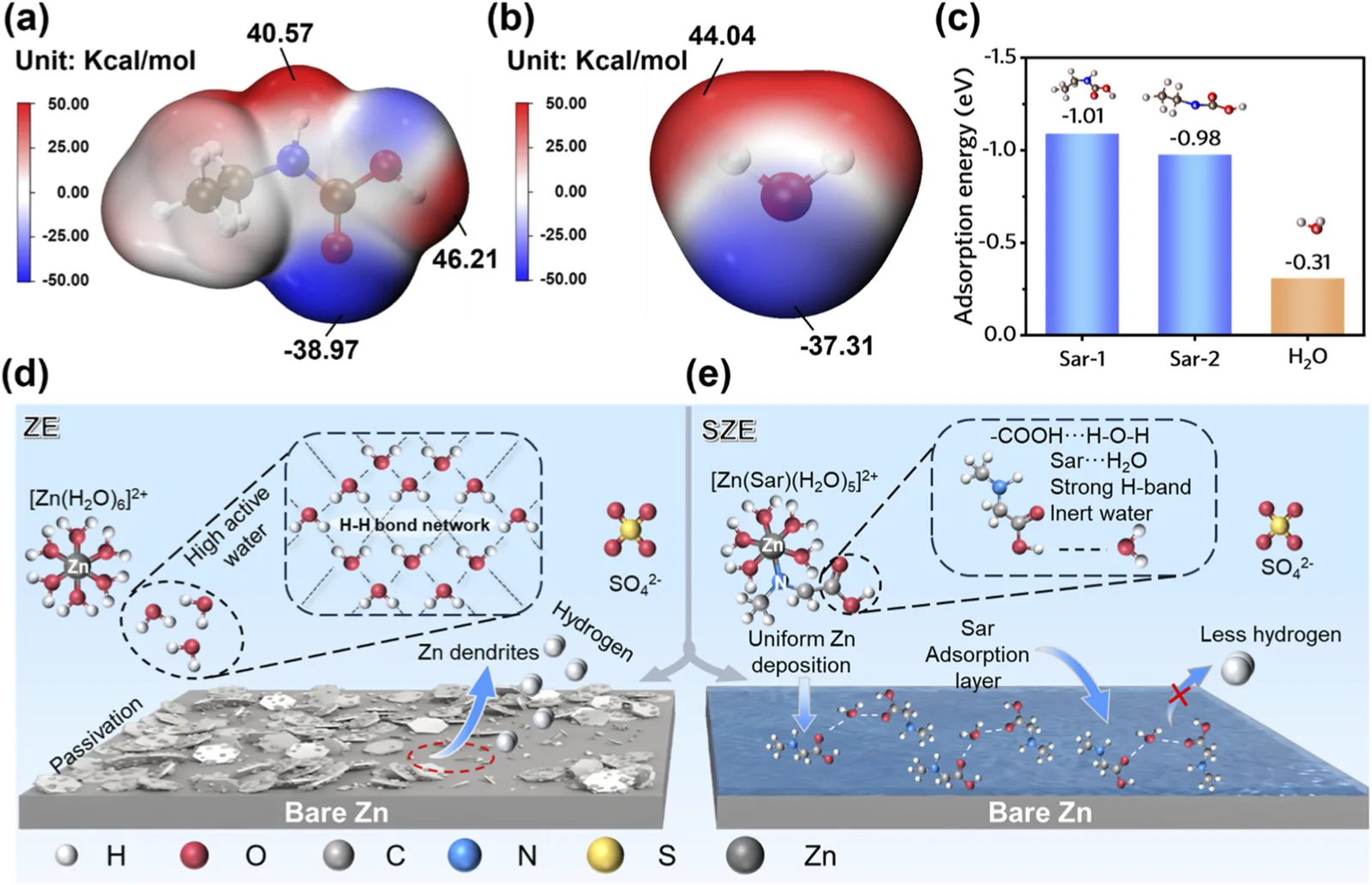
**(a)** Electrostatic potential distribution of the Sar molecule. **(b)** Electrostatic potential distribution of the H_2_O molecule. **(c)** Adsorption energies of Sar and H_2_O on the Zn (002) surface. Schematic illustration of the interfacial regulation mechanism of Sar additive in aqueous ZnSO_4_ electrolyte. **(d)** ZE (2 M ZnSO_4_) electrolyte induces severe hydrogen evolution, passivation, and Zn dendrite growth. **(e)** SZE reduces water activity through strong hydrogen-bond interactions, suppresses hydrogen evolution for uniform Zn deposition.

Molecular Dynamics (MD) simulations were carried out to study the solvation structure of Zn^2+^ in ZE (ZnSO_4_ electrolyte) and SZE (Sar-containing ZnSO_4_ electrolyte). In ZE, Zn^2+^ was mainly coordinated by the oxygen atoms of H_2_O, and the coordination numbers of Zn^2+^–H_2_O and Zn^2+^–SO_4_^2‒^ were 5.76 and 0.23, respectively (Supplementary Figure S1). After adding Sar, the coordination number of Zn^2+^–O(H_2_O) is slightly reduced to 5.74; that of Zn^2+^–O (SO_4_^2‒^) decreases to 0.20; and a weak Zn^2+^–O (Sar) coordination with a coordination number of 0.06 appears (Supplementary Figure S2). Based on the above results, Sar does not significantly substitute for H_2_O in the primary Zn^2+^ solvation shell; rather, weak coordination and hydrogen bonding are involved in regulating the local electrolyte environment. Moreover, the adsorption energies of Sar on Zn (002) are calculated to be −1.01 and −0.98 eV for two representative adsorption configurations, which are much lower than that of H_2_O on Zn (002) (−0.31 eV) (Figure 1c; Supplementary Figure S3). The stronger adsorption of Sar indicates that it can preferentially occupy the Zn surface and competitively suppress the direct adsorption/reduction of active water molecules. Therefore, Sar can synergistically regulate the electrolyte microenvironment and Zn anode interface through polarity-driven interactions, weak Zn^2+^ coordination, hydrogen-bond regulation, and preferential surface adsorption, thereby suppressing water-induced side reactions and promoting uniform Zn deposition.

As illustrated in Figure 1d, in a conventional aqueous electrolyte, zinc ions (Zn^2+^) predominantly form a [Zn(H_2_O)_6_]^2+^ hydration structure where the inner solvation sheath is entirely occupied by water molecules, confining SO_4_^2‒^ anions to the secondary sheath or bulk solution. The resulting abundance of highly active free water and the continuous hydrogen-bonding network trigger severe parasitic reactions. Specifically, the reduction of coordinated and free water at the anode leads to the HER, which causes battery gassing, electrolyte consumption, and the formation of an inhomogeneous passivation layer that increases interfacial impedance. Furthermore, incomplete desolvation coupled with an uneven interfacial electric field drives Zn to preferentially grow at local protrusions, forming dendrites that ultimately lead to internal short circuits. Conversely, Figure 1e shows that adsorbed sarcosine induces interfacial restructuring primarily through its strong anchoring at the Zn surface, synergistically coupled with hydrogen-bond water regulation. The surface-adsorbed sarcosine molecules anchor at the Zn anode interface and form strong intermolecular hydrogen bonds with ambient water molecules. This interaction disrupts the continuous hydrogen-bond network of free water, immobilizing active water into stable Sar–H_2_O assemblies and significantly suppressing the water-induced hydrogen evolution reaction and interfacial side reactions. Moreover, the interfacial adsorbed sarcosine modulates the electrode microenvironment to promote uniform Zn^2+^ deposition. Such regulation homogenizes interfacial ion flux, avoids localized concentrated deposition, and effectively inhibits the nucleation and growth of Zn dendrites. Benefiting from the interfacial adsorption effect of sarcosine and the synergistic hydrogen-bond modulation, the Zn anode achieves highly reversible plating/stripping and improved electrochemical stability.

Different concentrations (0.1 M, 0.5 M, 1 M) of Sar were added to the ZE. The mixtures were stirred for 2 h to achieve full dissolution and uniform mixing, and labelled SZE-0.1, SZE-0.5 and SZE-1. To investigate the regulation effect of Sar additive on the hydrogen-bond network of the electrolyte, Raman spectroscopy, FTIR spectroscopy, and NMR spectroscopy were performed. The Raman measurements (Figure 2a; Supplementary Figure S4) show that all four electrolytes exhibit a broad peak at 3,000–3,700 cm^‒1^ assigned to the O–H stretching vibration of water molecules.

**FIGURE 2:**
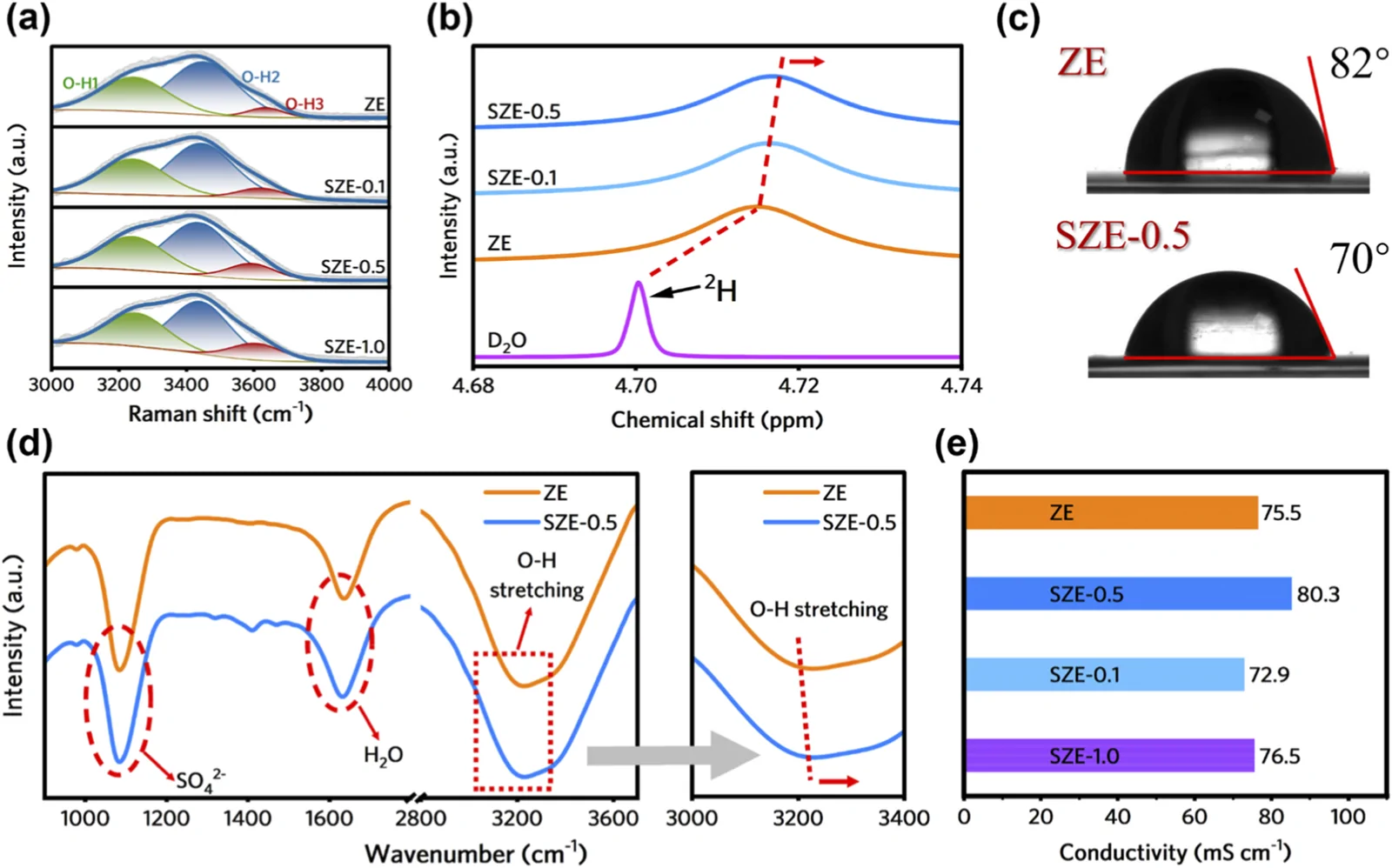
**(a)** Raman spectra showing the proportions of different hydrogen-bonded species in ZE and SZE; **(b)** ^2^H Nuclear Magnetic Resonance (NMR) spectra of D_2_O and different electrolytes; **(c)** contact angles of ZE and SZE-0.5; **(d)** the Fourier Transform Infrared Spectroscopy (FTIR)spectra of ZE and SZE electrolytes; **(e)** Corresponding ionic conductivities of the four different electrolyte systems.

After deconvolution, three types of water can be distinguished: O–H1 at 3,230 cm^‒1^ corresponding to strongly hydrogen-bonded water (free/active water), O–H2 at 3,450 cm^‒1^ corresponding to weakly hydrogen-bonded water, and O–H3 at 3,620 cm^‒1^ corresponding to non-hydrogen-bonded water (restricted by the additive and unable to participate in the continuous hydrogen-bond network). The pristine ZE electrolyte possesses the highest proportion of O–H1. With increasing Sar concentration, the fraction of O–H1 gradually decreases while that of O–H3 increases. This is attributed to the strong electronegativity of oxygen atoms in the carboxyl group (-COOH) of Sar, which forms strong hydrogen bonds (-COOH … H–O–H) with water molecules, disrupting the water–water strong hydrogen-bond network and converting highly active O–H1 into inert O–H3, thereby fundamentally suppressing the HER and anode corrosion. Moreover, the NMR characterization (Figure 2b) shows that the ^2^H signal of neat D_2_O appears at 4.702 ppm, and shifts to 4.715 ppm after ZnSO_4_ addition. This shift originates from strong coordination interactions between Zn^2+^ and D_2_O, which lowers the proportion of free water molecules in the system. With the introduction of Sar, the ^2^H peak further shifts downfield to 4.72 ppm in the electrolyte with 0.5 M Sar, confirming the continuous reduction of free water and the strong interaction between Sar and water molecules, providing supplementary evidence for hydrogen-bond network reconstruction and HER suppression. The regulation of the Zn anode interface and physicochemical properties of the electrolyte by Sar further supports its optimization effect on electrochemical performance. The contact angle decreased with increasing Sar concentration, and the SZE-0.5 electrolyte exhibited a smaller contact angle of 70° than that of ZE electrolyte (82°), demonstrating the enhanced wettability of the Sar-containing electrolyte on the Zn surface (Figure 2c). This improved surface affinity suggests that Sar-containing electrolyte can better wet the Zn anode, lower the Zn/electrolyte interfacial free energy, and promote uniform Zn deposition, which is beneficial for suppressing dendrite growth and interfacial side reactions.

Additionally, the FTIR spectra further verify the regulatory effect of Sar on the electrolyte environment. As shown in Figure 2d, the O–H stretching vibration of water molecules in the range of 3,100–3,600 cm^‒1^ shifts to higher wavenumbers after the introduction of Sar, indicating that the original hydrogen-bond network of water is weakened. Consistently, the H–O–H bending vibration in the range of 1600–1700 cm^‒1^ also exhibits a blue shift in the SZE-0.5 (0.5 M Sar-containing ZnSO_4_ Electrolyte) (Supplementary Figure S5a), further suggesting the interaction between Sar and water molecules and the reduced activity of free water. This hydrogen-bond regulation is beneficial for suppressing water-induced hydrogen evolution and corrosion reactions at the Zn anode interface. Meanwhile, the characteristic ν(SO_4_^2‒^) vibration peak shifts from 1081.6 cm^‒1^ in ZE to 1084.3 cm^‒1^ in SZE-0.5 (Supplementary Figure S5b), indicating that the interaction between Zn^2+^ and SO_4_^2‒^ is weakened after adding Sar.

Collectively, the above FTIR results show that Sar can regulate the water activity and ion-pair interaction in the electrolyte to suppress hydrogen evolution, corrosion and other side reactions. Optical photographs show that the Sar-containing electrolytes have been unchanged over time. As shown in Supplementary Figure S6, all electrolytes remained clear and transparent after 1 month of storage at room temperature; there was no visible precipitation or colour change, indicating good solubility and compatibility of Sar in the aqueous ZnSO_4_ electrolyte. In addition, calculated ionic conductivity (Figure 2e) combined with EIS measurements (Supplementary Figures S7,S8) show that the ionic conductivity of the electrolyte is increased to a certain extent after adding Sar, enhances the transport speed of Zn^2+^ and SO_4_^2‒^, lowers interfacial transport resistance, and maintains stable and high-performance battery operation.

To further verify the inhibition mechanism of Sar additive on side reactions of Zn anodes from the crystal structure level, XRD tests were performed on the cycled Zn anodes, and the results are shown in Figure 3a. Obvious characteristic peaks of byproducts appeared on the Zn anode cycled in the baseline electrolyte. In contrast, no byproduct peaks were observed on the Zn anode cycled in the modified electrolyte. Essentially, Sar forms strong hydrogen bonds with water molecules through its carboxyl groups to lock active water, while weakening the direct interaction between SO_4_^2‒^ and Zn^2+^ via coordination competition of its amino groups with Zn^2+^, thereby fundamentally inhibiting the corrosion reaction between Zn and water and the generation of SO_4_^2‒^-related byproducts.

**FIGURE 3:**
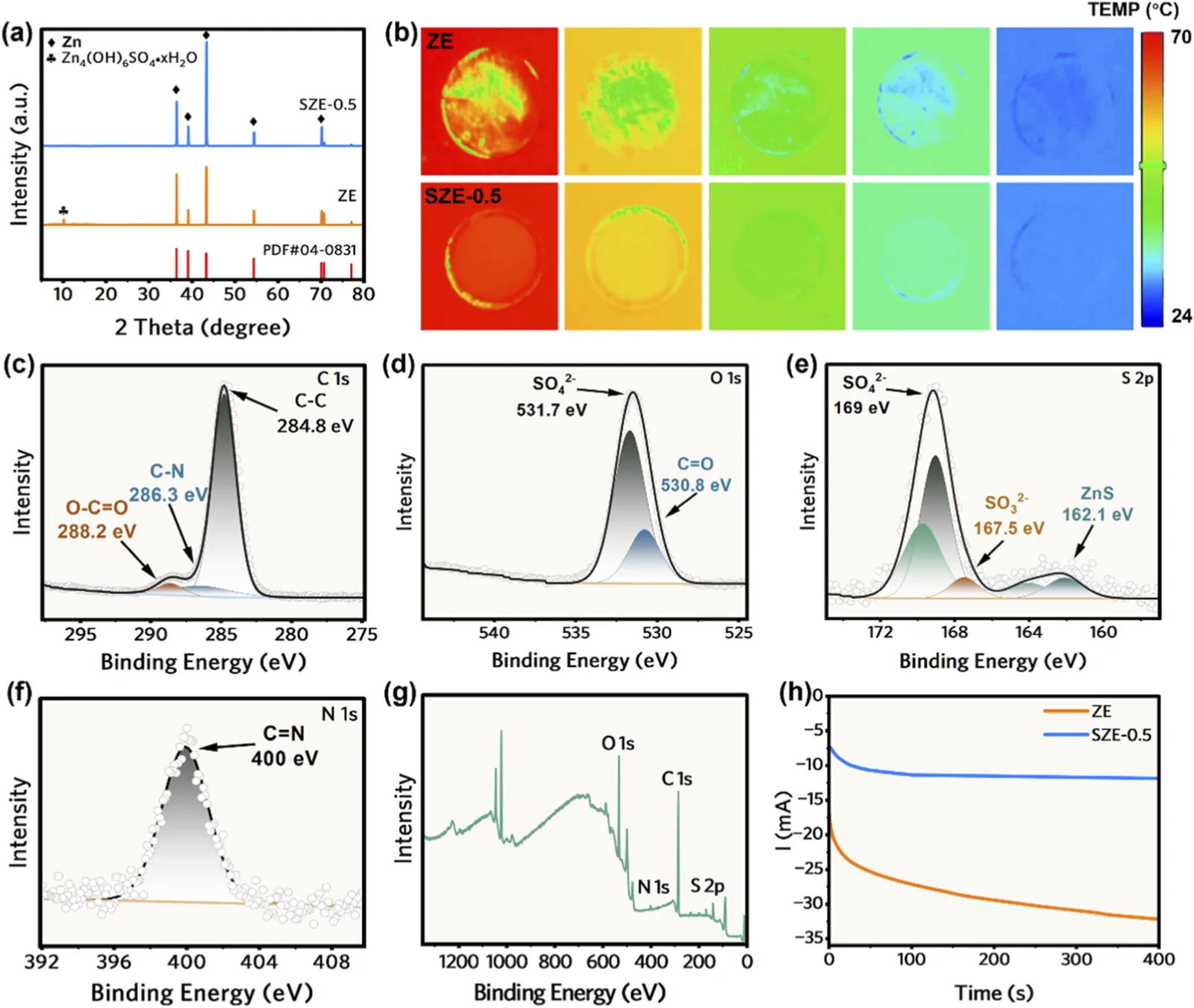
**(a)** XRD patterns of Zn anodes after 100 cycles at 1 mA cm^-2^ and 1 mAh cm^‒2^ in ZE and SZE-0.5 electrolytes; **(b)** Infrared thermal imaging images of the Zn anode surface **(c–g)** XPS spectra of the Zn anode after 20 cycles at 1 mA cm^-2^ and 1 mAh cm^‒2^ in the SZE-0.5 electrolyte: **(c)** C 1s, **(d)** O 1s, **(e)** S 2p, **(f)** N 1s, and **(g)** survey spectrum; **(h)** the Chronoamperometry (CA) test of ZE and SZE-0.5 electrolytes.

To further corroborate the interfacial optimization by Sar, infrared thermal imaging was used to characterize the Zn anodes after 100 cycles at 1 mA cm^-2^ and 1 mAh cm^‒2^ in ZE and SZE-0.5 electrolytes, as shown in Figure 3b. As can be seen, the Zn anode cycled in SZE-0.5 exhibits a smooth and flat surface without obvious irregular morphology or corrosion traces; while the Zn anode cycled in the baseline electrolyte shows significant irregular morphology, indicating severe corrosion and a large amount of by-product formation. To clarify the adsorption behaviour of Sar additive on the Zn anode surface and further reveal the essence of its interfacial protection, XPS tests were carried out on the Zn anode after 20 cycles at 1 mA cm^-2^ and 1 mAh cm^‒2^, and the results are shown in Figure 3c-g. Distinct characteristic peaks corresponding to C–N bonds and O–C=O groups are observed in the C 1s and N 1s spectra, verifying that Sar molecules and their derivatives are uniformly dispersed on the surface of the Zn anode to construct a functional electrode interfacial layer. This behavior arises from the preferential and stable adsorption of Sar molecules on the zinc anode: amino groups can coordinate with Zn^2+^, while carboxyl groups possess polar adsorption properties, and the synergy of these two effects promotes the generation of this functional interfacial layer. The SO_4_^2‒^ signal observed in the O 1s spectrum is presumed to be a slight precipitation of zinc salts from the electrolyte, which does not significantly affect the interfacial stability. In the S 2p spectrum, not only the characteristic peak of SO_4_^2‒^ (169.0 eV) but also the characteristic peak of ZnS (162.1 eV) and the SO_3_^2‒^ peak is detected, indicating chemical transformation of sulfur-containing species within the Sar-adsorbed interfacial layer.

In addition, the CA test results (Figure 3h) further reveal the regulatory effect of Sar additive on the Zn deposition process. In the ZE electrolyte, the deposition current of the Zn anode increases continuously with time, indicating that new active sites are continuously formed on the electrode surface during deposition, Zn dendrites grow continuously, leading to an increase in the electrode specific surface area and poor interfacial stability. In contrast, in the SZE-0.5 electrolyte system, the current rapidly stabilizes after zinc deposition nucleation without an obvious continuous rising trend, demonstrating that the zinc deposition process is uniform and controllable, and dendrite growth is effectively suppressed. The above results reveal that Sar molecules uniformly adsorb on the Zn anode surface, forming a stable adsorbed interfacial layer. This layer isolates active water from the Zn electrode, suppresses corrosion and side reactions, induces homogeneous and compact Zn^2+^ deposition, and strengthens Sar’s regulation on interfacial stability.

To intuitively demonstrate the inhibitory effect of the Sar electrolyte additive on dendrite growth and hydrogen evolution, *in situ* optical microscopy was employed to monitor the morphology evolution of the Zn anode during plating/stripping at a current density of 10 mA cm^-2^. As shown in Figure 4a, after 5 min of Zn plating in the baseline ZE electrolyte, numerous black and loose deposits appeared on the Zn foil surface and gradually became more severe with prolonged deposition, accompanied by obvious gas bubble evolution. In marked contrast, the Zn foil cycled in SZE-0.5 retained a relatively smooth, flat surface throughout Zn plating/stripping, with no visible gas bubbles. Therefore, Sar reduces the growth of dendritic Zn and hydrogen evolution and promotes more uniform Zn deposition. To further examine the shape of the deposited material, cycled Zn anodes were tested after 100 cycles in Zn||Zn symmetric cells at 1 mA cm^-2^ and 1 mAh cm^‒2^. As displayed in Figure 4b-c, the Zn anode cycled in ZE exhibited massive two-dimensional sheet-like deposits stacked on the surface, whereas the Zn anode cycled in SZE-0.5 showed a much smoother and more compact surface without obvious dendrites or corrosion.

**FIGURE 4:**
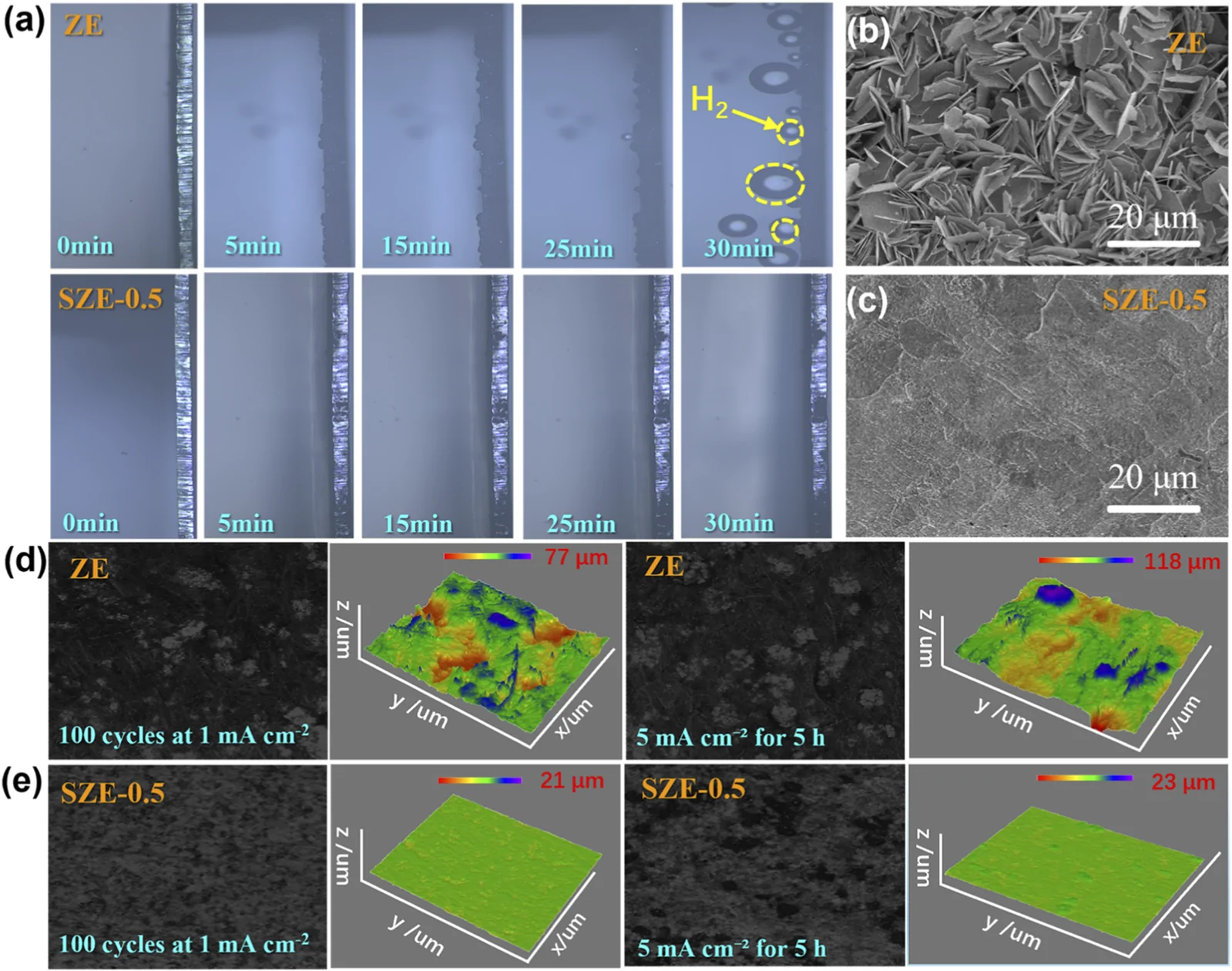
**(a)** *In-situ* optical microscopy images of Zn deposition on 100-μm-thick Zn foil at a current density of 10 mA cm^-2^ in ZE and SZE-0.5 electrolytes **(b,c)** SEM images of Zn anodes after 100 cycles at 1 mA cm^-2^ and 1 mAh cm^‒2^ in ZE and SZE-0.5 electrolytes **(d,e)** Confocal laser scanning microscopy (CLSM) images and corresponding 3D reconstructed images of Zn anodes under different conditions in ZE and SZE-0.5 electrolytes.

The suppressed hydrogen evolution was further supported by the thickness variation of Zn||Zn symmetric cells after cycling under different conditions. As shown in Supplementary Figure S9, the initial cell thickness was 2.976 mm. After 100 cycles at 1 mA cm^-2^ and 1 mAh cm^‒2^, the thickness of the cell using ZE increased to 3.138 mm, while that of the cell using SZE-0.5 was only 3.067 mm. At a higher current density of 5 mA cm^-2^ with the same areal capacity of 1 mAh cm^‒2^, the ZE-based cell further expanded to 3.171 mm, whereas the SZE-0.5-based cell showed a smaller thickness of 3.082 mm. The reduced thickness increase in SZE-0.5 indicates that Sar effectively mitigates gas accumulation and cell swelling during repeated Zn plating/stripping, further confirming its role in suppressing hydrogen evolution and stabilizing the Zn/electrolyte interface.

The corrosion resistance of Zn foils in different electrolytes was further evaluated by XRD after soaking for 7 days. As shown in Supplementary Figure S10, distinct diffraction peaks corresponding to Zn(OH)_6_SO_4_·xH_2_O were observed on the Zn foil soaked in the pristine ZE electrolyte, indicating the occurrence of serious interfacial corrosion and parasitic side reactions. In contrast, the Zn foils soaked in Sar-containing electrolytes showed no obvious byproduct peaks, and only the characteristic peaks of metallic Zn were detected. These results demonstrate that the introduction of Sar effectively suppresses the formation of corrosion byproducts and enhances the chemical stability of the Zn anode in the aqueous electrolyte. In addition, *in situ* 3D laser confocal scanning microscopy (LCSM) was used to characterize the surface of cycled and plated Zn anodes. Figures 4 d-e show the surface morphologies and corresponding 3D reconstructed images of Zn anodes after 100 cycles at 1 mA cm^-2^ and 1 mAh cm^‒2^, as well as after plating for 5 h at 5 mA cm^-2^ in ZE and SZE-0.5 electrolytes, respectively. The maximum surface heights in the baseline electrolyte were approximately 77 μm and 118 μm, whereas those in the SZE-0.5 functional electrolyte were only 21 μm and 23 μm, demonstrating that the surface flatness and uniformity of the Zn anode were significantly improved in SZE-0.5. This further confirms that the Sar-containing electrolyte additive facilitates uniform deposition of metallic Zn and suppresses side reactions at the anode.

To further elucidate how Sar regulates the Zn anode interface, we examined Zn deposition behavior, interfacial reaction kinetics, and properties of the electrode/electrolyte interface. These analyses were used to assess the combined effects of Sar on Zn deposition, reaction barriers, and interfacial charge-transfer behavior. Zn nucleation and growth in ZE and SZE-0.5 were compared using Zn||Cu asymmetric cells (Figures 5a, b). In the modified electrolyte system with 0.5 M Sar additive added, the nucleation overpotential for growth gradually levels off as the areal capacity increases. By contrast, in the baseline electrolyte system, the nucleation overpotential remains high with increasing areal capacity and shows no levelling-off trend.

**FIGURE 5:**
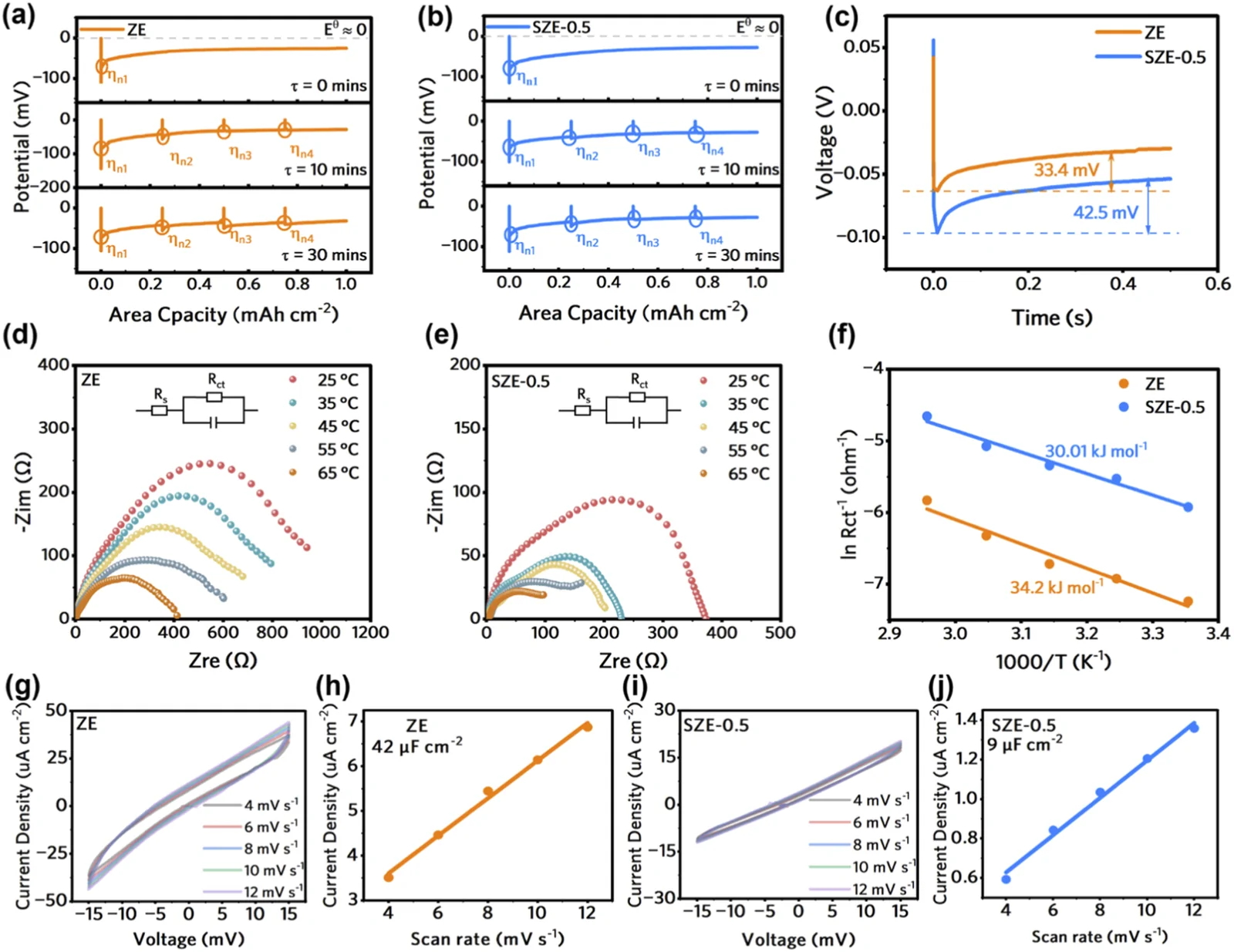
Relevant test results of Zn||Cu asymmetric cells assembled with ZE and SZE-0.5 electrolytes: **(a,b)** Zn deposition-nucleation overpotential curves in ZE and SZE‐0.5 electrolytes, respectively; **(c)** Comparison of nucleation overpotentials between the two electrolytes; **(d,e)** Electrochemical impedance spectroscopy (EIS) of ZE and SZE-0.5 electrolytes at different temperatures, respectively; **(f)** Comparison of reaction activation energies of the two electrolytes; Cyclic voltammetry (CV) curves at different scan rates and corresponding double-layer capacitance (Cdl) results of Zn||Zn symmetric cells in the voltage range of −15–15 mV: **(g,h)** ZE electrolyte system; **(i,j)** SZE-0.5 electrolyte system.

From the potential-time curve of single deposition (Figure 5c), the introduction of Sar increased the Zn nucleation overpotential from 33.4 mV to 42.5 mV. A higher nucleation overpotential favors the formation of uniformly distributed nuclei and refined grain deposition, providing a kinetic basis for suppressing dendrite growth.

Meanwhile, the kinetic behaviors of Zn deposition/dissolution in ZE and SZE-0.5 electrolytes were compared via temperature-dependent electrochemical impedance spectroscopy (EIS) and Arrhenius activation energy calculation. As displayed in Figures 5d, e and Supplementary Table S1, although the charge-transfer resistance (Rct) of the Zn anode in ZE decreased with rising temperature, the overall interfacial reaction resistance remained high. In SZE-0.5, however, Rct at the same temperature was remarkably lowe r, and its decline with temperature became milder, suggesting that Sar effectively reduced the charge-transfer resistance during Zn deposition/dissolution and accelerated interfacial reaction kinetics. Activation energy calculated by Arrhenius fitting (Figure 5f) showed that the energy barrier for Zn deposition in ZE was 34.2 kJ mol^-1^, whereas that in SZE-0.5 decreased to 30.01 kJ mol^-1^. The lower activation energy verified that the energy barrier of Zn^2+^ reduction was significantly weakened, which is consistent with EIS results. Together, they confirm that Sar enables highly efficient and reversible interfacial reactions by lowering the activation energy and accelerating charge-transfer kinetics. Furthermore, as shown in Figures 5g, i, within a small potential window of −15 to 15 mV, CV curves of both electrolytes exhibited typical capacitive behavior without obvious redox peaks, and the current density increased linearly with scan rate. Linear fitting of current density difference versus scan rate (Figures 5h, j) yielded a double-layer capacitance (Cdl) of 42 μF cm^-2^ for ZE, whereas Cdl in SZE-0.5 sharply decreased to 9 μF cm^-2^. The greatly reduced Cdl reveals that Sar can selectively adsorb on the Zn surface, effectively covering high-activity sites, inhibiting water decomposition and other side reactions, regulating interfacial charge distribution, and optimizing the Zn^2+^ deposition environment, thus providing essential interfacial support for uniform and stable Zn deposition.

The Tafel polarization measurements were used to assess the corrosion behavior and interfacial reaction kinetics of the Zn anode (Figure 6a). The corrosion current density in ZE was 5.42 mA cm^-2^ and in SZE-0.5 it was 1.11 mA cm^-2^, so it had dropped by about 80%. At the same time, the corrosion potential increased from −0.9272 V in ZE to −0.8995 V in SZE-0.5. Together, the lower corrosion current density and more positive corrosion potential were in line with reduced Zn corrosion in the Sar-containing electrolyte. A change in the slope of polarisation concurrently occurred, thus, the reaction kinetics at the Zn/electrolyte interface had changed.

**FIGURE 6:**
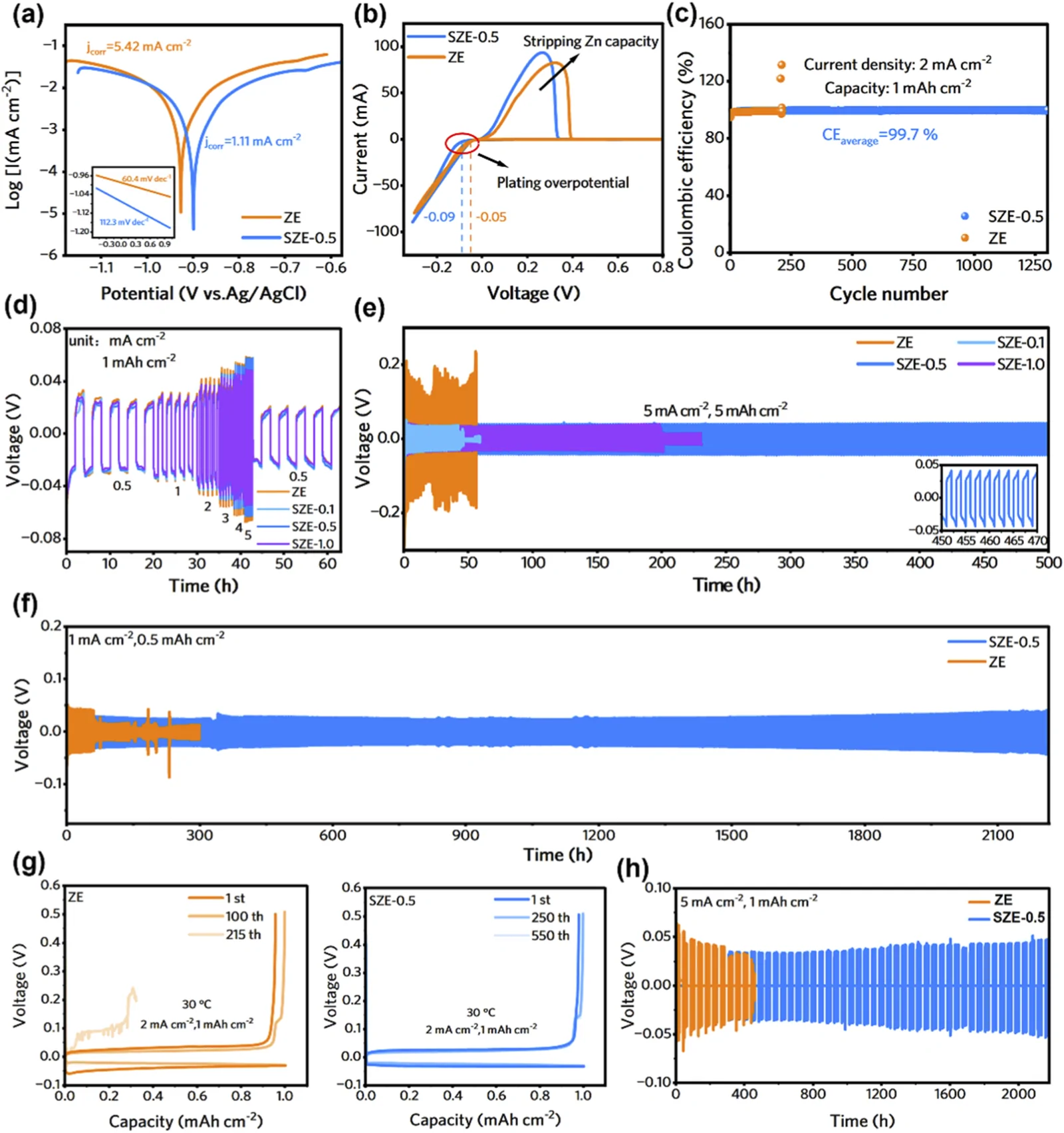
**(a)** Tafel curves of Zn anodes after 100 cycles at 1 mA cm^-2^ and 1 mAh cm^‒2^ in ZE and SZE-0.5 electrolytes; **(b)** CV curves of Zn||Cu asymmetric cells assembled with ZE and SZE-0.5 electrolytes; **(c)** Coulombic efficiency of Zn||Cu asymmetric cells assembled with ZE and SZE-0.5 electrolytes; Relevant test results of Zn||Zn symmetric cells assembled with ZE and SZE-0.5 electrolytes: **(d)** Rate performance curves; **(e)** Cycling performance at 5 mA cm^-2^ and 5 mAh cm^‒2^; **(f)** Cycling performance at 1 mA cm^-2^ and 0.5 mAh cm^‒2^; **(g)** Charge-discharge curves of Zn||Cu asymmetric cells assembled with ZE and SZE-0.5 electrolytes at 2 mA cm^-2^ and 1 mAh cm^‒2^; **(h)** Calendar life test results of Zn||Zn symmetric cells assembled with ZE and SZE-0.5 electrolytes at 5 mA cm^-2^ and 1 mAh cm^‒2^.

The Tafel plots for HER show that the slope has decreased from 112.3 mV·dec^−1^ in ZE to 60.4 mV·dec^−1^ in SZE-0.5 (Figure 6a, inset). Linear Sweep Voltammetry (LSV) experiments provided stronger support for the delayed onset of HER (Supplementary Figure S11). At both 0.5 M and 1.0 M concentrations of Sar, the cathodic sweep showed a more negative HER onset potential than ZE. SZE-0.5 had the largest shift, moving from −1.073 V in ZE to −1.244 V, and was cathodic by 171 mV. Therefore, a larger cathodic polarisation was needed to induce HER and thus resulted in delayed hydrogen evolution in SZE-0.5. In short, the above results indicate that Sar suppresses Zn corrosion and delays the start of HER, thus enhancing the interface stability of the Zn anode. CV was used to investigate the impact of Sar on Zn plating/stripping behaviour further (Figure 6b). After adding the Sar, the Zn deposition potential shifted to a more negative value of −0.09 V. This change may be due to an increased nucleation overpotential, and thus more small, evenly distributed Zn nuclei will be formed. Thus, the nucleation pattern will restrict local growth of coarse deposits and enhance uniformity of Zn deposition. The Zn-stripping oxidation peak current was also relatively high in SZE-0.5 compared with ZE, and the symmetry of the oxidation and reduction peaks was greater. The above modifications have improved the reversibility of Zn plating/stripping and shown more stable interfacial electrochemical behaviour. Figure 6c is the Coulombic efficiency profile for Zn||Cu asymmetric cells at 2 mAcm^‒2^ and 1 mAhcm^‒2^. ZE had a large fluctuation in Coulombic efficiency and could only be stably cycled for about 200 cycles. SZE-0.5 cells, on the other hand, had a relatively high mean Coulombic efficiency of 99.7% and maintained stable operation for more than 1,200 cycles. Based on the above analysis, there will be reduced parasitic reactions at the Zn anode and enhanced reversibility of Zn plating/stripping; thus, the cycle life will be extended significantly.

To evaluate the rate capability of the Zn anode, Zn||Zn symmetric cells were tested at various current densities (Figure 6d). Across 0.5–5 mAcm^‒2^, the polarization voltage in ZE rose sharply with current density and showed significant fluctuations. This behavior was in line with unstable Zn plating/stripping at the electrode/electrolyte interface. Sar addition reduced the polarization voltage, and increasing the Sar concentration to 0.5 M led to a further reduction. Therefore, SZE-0.5 had a smoother and more stable voltage curve in the rate test and exhibited better rate capability and interfacial stability. Electrolytes of 0.5 M and 1.0 M Sar show relatively low polarisation and good interfacial stability under high current density. Furthermore, at a current density of 0.5 mA cm^-2^, the polarization voltage also fully recovers; thus, Sar can effectively suppress dendrite growth and side reactions to extend the rate performance and interfacial reversibility of the Zn anode. Figure 6e is the cycle stability of Zn||Zn symmetric cells at a high current density of 5 mA cm^-2^ and a high areal capacity of 5 mAh·cm^‒2^. The Zn anode in ZE has a wide and violently fluctuating polarisation window, and fails after about 50 h. Add 0.1 M of Sar; although it slightly improves the interface, the cycle life is still short. The 1.0 M Sar system will have a life of about 200 h. On the other hand, SZE-0.5 (0.5 M Sar) has better performance, a narrow and stable polarisation window, and stable cycling for over 500 h. Additionally, as shown in the long-cycle GCD curves in Fig. 6f, at 1 mA cm^-2^ and 0.5 mAh·cm^‒2^, the Zn anode of SZE-0.5 has a narrow and stable window and can cycle more than 2000 h, thus further demonstrating the optimization effect of Sar on interfacial stability. Notably, the voltage (overpotential) of the cells exhibits an initial decrease during the early cycling stage, which can be ascribed to an activation process. In the initial cycles, the freshly polished Zn surface is not yet fully covered by the adsorbed sarcosine (Sar) layer, and the electrolyte has not completely wetted the electrode/separator interface, resulting in a relatively high nucleation overpotential. With prolonged cycling, Sar gradually adsorbs onto the Zn surface and homogenizes the Zn^2+^ flux, and the electrolyte fully penetrates the interface, so the overpotential decreases and then stabilizes at a low and constant value.

Additionally, Figure 6g presents charge-discharge curves of Zn||Cu asymmetric cells at 2 mA cm^-2^ and 1 mAh·cm^‒2^ at selected cycles. In ZE, the polarization increases noticeably with cycling, the charging plateau fluctuates significantly, and the curve is severely distorted with drastic capacity fading at the 215th cycle, indicating extremely poor interfacial stability. In SZE-0.5, however, the charge-discharge curves at the 1st, 250th, and 550th cycles almost overlap completely, the polarization remains low, the capacity is stable, and no distortion is observed, fully demonstrating that Sar enables durable interfacial stability of the Zn anode. Figure 6h shows the calendar life test curves of Zn||Zn symmetric cells at 5 mA cm^-2^ and 1 mAh·cm^‒2^. The results reveal that in SZE-0.5, the Zn anode possesses a narrow and smooth polarization window and can operate stably for over 2000 h. This further confirms that the Sar additive effectively inhibits Zn dendrite growth and side reactions even at high current densities, significantly enhancing the interfacial stability and long-term cycle life of the Zn anode, in good agreement with the above characterizations.

To assess the applicability of Sar in full-cell configurations, Zn||ZnI@CNT-AC cells were assembled using the previously described ZnI@CNT-AC positive electrode and Zn negative electrode. Cells with ZE or SZE-0.5 were used in the same experiments. Figure 7a compares the CV responses of the full cells with ZE and SZE-0.5 electrolytes. The reduction peak shifts from 1.183 V in ZE to 1.194 V in SZE-0.5. Notably, the elevated anodic current near 1.6 V in the SZE-0.5 sample is an irreversible current tail originating from high-potential side reactions (most likely electrochemical oxidation of adsorbed sarcosine), rather than a reversible redox peak associated with the charge-storage process. This irreversible process only occurs at high potential and does not participate in the reversible redox reaction of the full cell. Figure 7b, c are the galvanostatic charge-discharge curves at different rates. SZE-0.5 had a higher specific charge and discharge capacity and less capacity fading at high rates than ZE. The charge-discharge plateaus also had a smaller voltage drop and were flatter and more stable. The above trends show less cell polarisation and a higher rate capability for SZE-0.5, which are consistent with a stable Zn/electrolyte interface.

**FIGURE 7:**
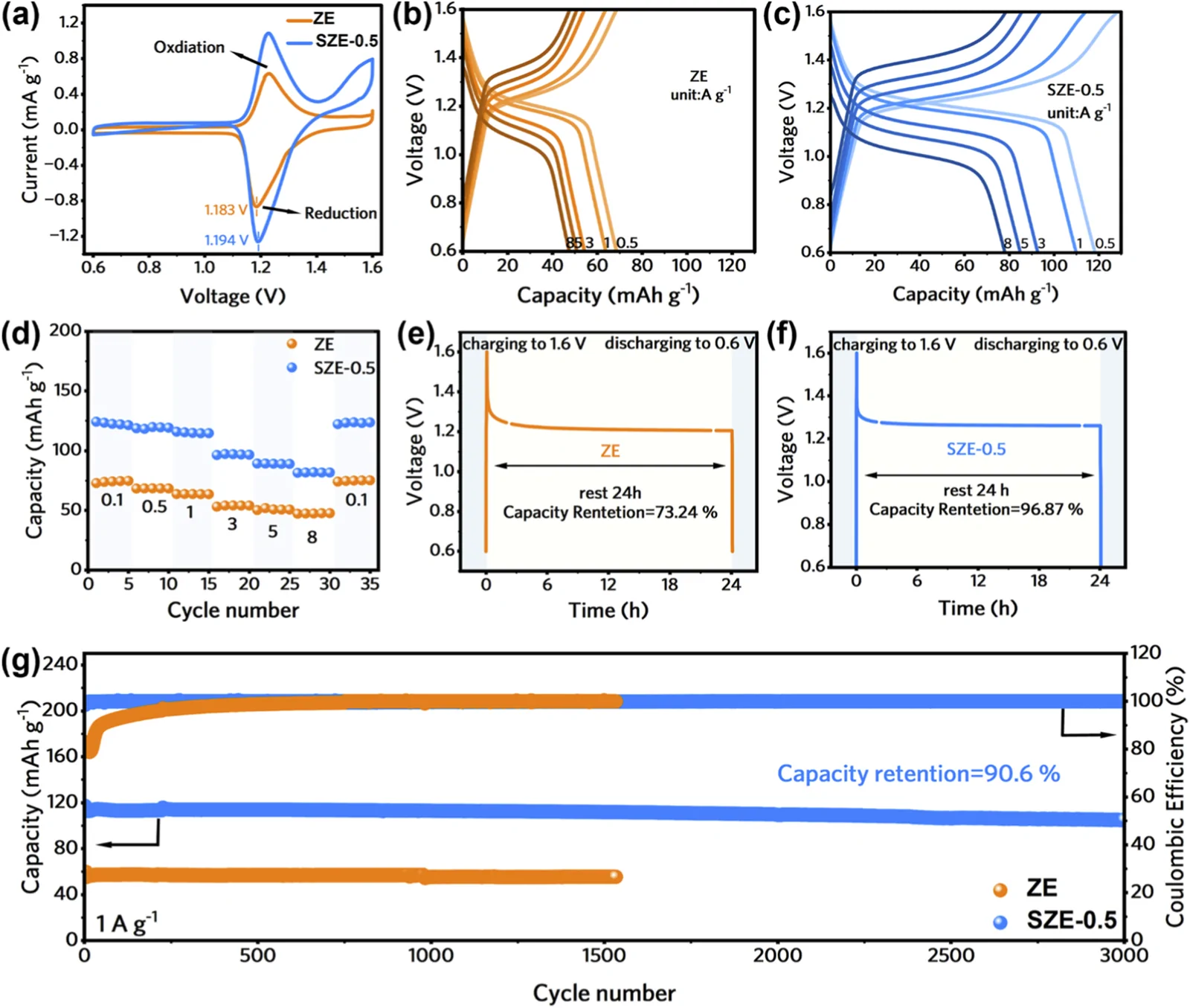
Relevant test results of Zn||ZnI@CNT-AC full cells assembled with ZE and SZE-0.5 electrolytes, which were used to investigate the stability and reversibility of Zn anodes in the two electrolytes: **(a)** CV curves. The slight current rise near the upper potential limit (∼1.6 V) corresponds to an irreversible anodic side reaction rather than a reversible redox process, and it does not contribute to the reversible battery capacity; **(b,c)** Charge-discharge curves; **(d)** Rate performance curves; **(e,f)** Self-discharge test curves; **(g)** Cycling performance curves.

Figure 7d shows the rate capability of the full cells. Under all tested specific currents, SZE-0.5 had a higher specific discharge capacity than ZE. When the specific current was reduced from 8 to 0.1 A g^-1^, the SZE-0.5 cell almost reached its original capacity. Recovery indicates a high rate of reversibility and is consistent with enhanced interfacial behaviour in the presence of Sar. Self-discharge behaviour is an essential practical indicator of aqueous zinc-ion batteries that reflects the loss of charge over time at rest and sensitivity to parasitic reactions. After 24 h of rest, the cell in ZE showed a significant drop in voltage and had lost about 26.76% of its capacity (Figure 7e). The cell in SZE-0.5 had a small voltage drop and retained 96.87% of its capacity (Figure 7f). In light of the previous corrosion results, this data indicates a reduction in Zn corrosion and fewer parasitic reactions; thus, there is improved charge retention during storage. Figure 7g shows the extended cycle performance of the full cells at 1 A⋅g^‒1^. The ZE cell cycled about 1,500 times and then showed significant capacity loss. SZE-0.5 had a higher initial specific discharge capacity and retained 90.6% of that capacity after 3,000 cycles, however. The Coulombic efficiency is almost 100% after many cycles. Therefore, the cycling stability and reversibility shown in the results can be attributed to Sar, as shown by the previous interfacial and mechanistic studies.

## 3 Conclusion

In conclusion, this study has developed a sarcosine-based electrolyte system that stabilizes Zn-metal anodes by modulating both the bulk water and the Zn/electrolyte interface via an adsorption-dominated mechanism. Spectroscopic measurements showed that Sar weakened the native water hydrogen-bond network, and first-principles calculations and interfacial analyses confirmed the preferential adsorption of Sar on Zn(002) and the formation of a dense adsorbed Sar layer at the interface. The combined effect of the above reduced water-induced hydrogen evolution and corrosion, suppressed inactive by-product generation, lowered the Zn^2+^-reduction barrier, and promoted uniform Zn nucleation and deposition. Therefore, SZE-0.5 enabled Zn||Zn cells to cycle for 2100 h and maintained an average Coulombic efficiency of 99.7% for more than 1200 cycles for Zn||Cu cells. After a 24 h rest, the Zn||ZnI@CNT-AC full cell retained 96.87% of its capacity and 90.6% after 3,000 cycles; the Coulombic efficiency remained close to 100%. Based on the above experiments, combining water-structure regulation with adsorption-dominated mechanism can be used to achieve reversible Zn electrochemistry. Further validation at a high electrode loading, lean-electrolyte, and limited Zn-excess will be needed to confirm its practicality.

## Data Availability

The original contributions presented in the study are included in the article/Supplementary Material, further inquiries can be directed to the corresponding authors.
