# Supplementary material for: Adsorption-dominated interfacial regulation by sarcosine via hydrogen-bond water modulation for stable Zn metal anodes
Source: Front Chem. 2026 Oct 2;14:1986660. doi: 10.3389/fchem.2026.1986660 (PMC13672593; doi:10.3389/fchem.2026.1986660)
Supplement: Supplementary file 1 [file DataSheet1.pdf]

Supporting Information

Adsorption-Dominated Interfacial Regulation by Sarcosine via Hydrogen-Bond Water Modulation for Stable Zn Metal Anodes

ZijingWang,[1] Haolin Ju,[2] Hao Zhang,[1] Yiqian Yang,[1] Huan Liu,[1*] Bin Cao,[1*] Jie Ding,[1] Bin Xu [2, 3*]

[1] College of Materials Science and Engineering, Xi'an University of Science and Technology, Xi'an 710054, China. E-mail: huanliu@xust.edu.cn (H. Liu)

[2] State Key Laboratory of Organic Inorganic Composites, Beijing Key Laboratory of Electrochemical Process and Technology for Materials, Beijing University of Chemical Technology, Beijing 100029, China. E-mail: caobin@xust.edu.cn (B. Cao)

[3] Institute of Advanced Energy Storage Materials and Technologies, School of Chemistry and Chemical Engineering, Yan'an University, Yan'an 716000, China.

E-mail: xubin@yau.edu.cn (B. Xu)

Experimental Section

Preparation of Zn Anodes

Metallic zinc foil with a thickness of 100 μm was used as the raw electrode material. First, the surface of the Zn foil was gently wiped with dust-free paper sprayed with anhydrous ethanol to remove surface dust and oil contamination. Subsequently, the Zn foil was cut into circular electrodes with a diameter of 12 mm using a disk cutter (MSK-110). The as-cut Zn disks were sonicated in anhydrous ethanol for 15 min to thoroughly remove residual impurities and surface oxide layers. After sonication, the Zn disks were dried in a vacuum oven for 2 h, then cooled to room temperature and stored for further use.

Preparation of Electrolytes

Both the baseline electrolyte and modified electrolytes were prepared using zinc sulfate heptahydrate ($ZnSO_4 \cdot 7H_2O$) as the solute and deionized water as the solvent. In detail, $ZnSO_4 \cdot 7H_2O$ was added into a 10 mL glass vial at a predetermined ratio and stirred at 400 rpm at 25±1 °C to form a 2 M $ZnSO_4$ baseline electrolyte (denoted as ZE). Thereafter, sarcosine (Sar) additive was introduced into the baseline electrolyte at various concentrations (0.1 M, 0.5 M, and 1 M), followed by continuous stirring for 2 h to ensure complete dissolution and homogeneous mixing. These samples were labeled SZE-0.1, SZE-0.5 and SZE-1. All electrolytes were sealed and stored in the dark at room temperature before use.

Preparation of ZnI@CNT-AC Cathodes

ZnI@CNT-AC cathodes were fabricated as follows. First, carbon nanotubes (CNTs) and activated carbon (AC, YP50F) were precisely weighed at a mass ratio of 1:9. Then, CNTs and AC were separately mixed with isopropanol at a mass ratio of 2:3 and sonicated individually for 20 min. The two dispersions were combined and sonicated for another 20 min to achieve uniform dispersion. The mixed dispersion was vacuum-filtered to form a free-standing flexible film, which was then dried in a vacuum oven at 60 °C for 8 h. After cooling to room temperature, the film was cut into circular

disks with a diameter of 10 mm. A ZnI_2–ethanol solution was uniformly drop-cast onto the as-prepared CNT-AC film, followed by further drying at 60°C for 8 h in a vacuum oven to obtain the final ZnI_2@CNT-AC cathode.

The solvation structure of the electrolyte was characterized using a Fourier-transform infrared (FTIR) spectrometer (Thermo Fisher Scientific, Nicolet iS20). Raman spectra were collected using a confocal micro-Raman spectrometer (LabRAM, HORIBA). The morphology of the samples was observed via field-emission scanning electron microscopy (FE-SEM; Hitachi, S-4800, 20 kV). The deposition/stripping process of the samples during cycling was monitored in real time using an in situ optical microscope (YUESCOPE). The crystal structure and composition of the samples were analyzed by X-ray diffraction (XRD; XPert Pro MPD, Panalytical).

Electrochemical Characterization

For the symmetric cells, asymmetric cells, and full cells assembled in CR2025 coin cells, zinc foils served as both the counter and reference electrodes, with Whatman glass fiber membranes selected as the separators. The electrolyte used was either $ZnSO_4$ or Sar/$ZnSO_4$ electrolyte, and zinc foils, copper foils, and ZnI@CNT-AC were utilized as the working electrodes, respectively. Prior to use, the zinc foils underwent ultrasonic cleaning. Linear sweep voltammetry (LSV) curves and Tafel plots were measured in the $ZnSO_4$ or Sar/$ZnSO_4$ electrolyte using a three-electrode system, where a Pt electrode, a Zn electrode, and an Ag/AgCl electrode were employed as the counter, working, and reference electrodes, respectively.

Density functional theory (DFT) calculations

The electrostatic potential (ESP), as well as the lowest unoccupied molecular orbital (LUMO) and highest occupied molecular orbital (HOMO) energy levels of Sar and H_2O were calculated by using the Gaussian 16 package.(Frisch et al., 2016) Geometry optimizations and single-point calculations for MENSA were performed at the B3LYP(Stephens et al., 1994) /6-311+G(d,p)(Krishnan et al., 1980) level. The DFT-D3 (Becke-Johnson) method(Grimme et al., 2010; Grimme et al., 2011) was included

to account for van der Waals interactions. The visualizations of the ESP, LUMO and HOMO energy level results were using the Multiwfn(Lu and Chen, 2012) and VMD(Humphrey et al., 1996) software.

Adsorption energies calculated using the projector-augmented wave (PAW) method as implemented in the Vienna Ab initio Simulation Package (VASP) software.(Kresse and Furthmüller, 1996) The plane-wave cutoff energy for all calculations was set to 500 eV. The Perdew–Burke–Ernzerhof (PBE) functional within the generalized gradient approximation (GGA)(Perdew et al., 1996) was used as the exchange-correlation functional, and the DFT-D3 (Grimme) method(Grimme et al., 2010) was employed to account for van der Waals interactions. For the adsorption energy (E_{ad}) calculations, the Zn(002) slab model is modeled by a four-layer 6×6 surface supercell, and a 25 Å vacuum layer was constructed along the z-direction. The convergence criterion for total energy was 10^{-5} eV, and the force on each atom was < 0.03 eV Å^{-1}. Γ-only k-meshes were used and all of the atoms were allowed for geometry relaxation. The adsorption energy was defined by the following equation:

$$E_{ad} = E_{Zn002\text{-}m} - E_{Zn002} - E_m$$

where $E_{Zn002\text{-}m}$ is the total energy of the slab models after adsorbing a H_2O or Sar molecule, E_{Zn002} is the energy of a free slab model, and E_m is the energy of H_2O or Sar molecule. Dipole corrections along the z-direction were also applied. The visualizations of the adsorption models were carried out using the VESTA(Momma and Izumi, 2008) software.

Molecular dynamics (MD) simulations

MD simulations for electrolytes were performed using the GROMACS simulation package(Abraham et al., 2015) with the all-atom optimized potential for liquid simulations (OPLS-AA)(Jorgensen et al., 1996). The OPLS-AA force field parameters for Sar, and water molecules were established by the Ligpargen web server.(Jorgensen

and Tirado-Rives, 2005; Dodda et al., 2017; Dodda et al., 2017) All the structures, including $ZnSO_4$, Sar, and water, were optimized by using the Gaussian 16 package at the 6-311+G(d, p) level. The DFT-D3 (Becke-Johnson) correction was also taken into account for the van der Waals interactions. Atomic charges were calculated using the restrained electrostatic potential 2 (RESP2) method via Gaussian and Multiwfn. The initial simulation box size was set to 5 nm × 5 nm × 5 nm. The MD simulation protocol comprised five sequential stages: two-stage energy minimization, two-stage re-equilibration, and a production simulation stage. Energy minimization was performed first using the steepest descent algorithm for 200,000 steps, followed by the conjugate gradient method for 20,000 steps. The system was then pre-equilibrated in the constant-temperature, constant-volume (NVT) ensemble at 298.15 K for 100 ps using the V-rescale thermostat. Subsequently, a constant-temperature, constant-pressure (NPT) ensemble with the V-rescale thermostat coupled with the Berendsen barostat was run at 298.15 K and 1.01325 bar for 2 ns. Finally, a production simulation was carried out in the NPT ensemble under the same conditions (V-rescale and Berendsen barostat) for 20 ns. A time step of 2 fs was used throughout, and the particle-mesh Ewald (PME) method(Darden et al., 1993) was employed. The visualizations of the MD simulations were obtained using the VESTA and VMD software.

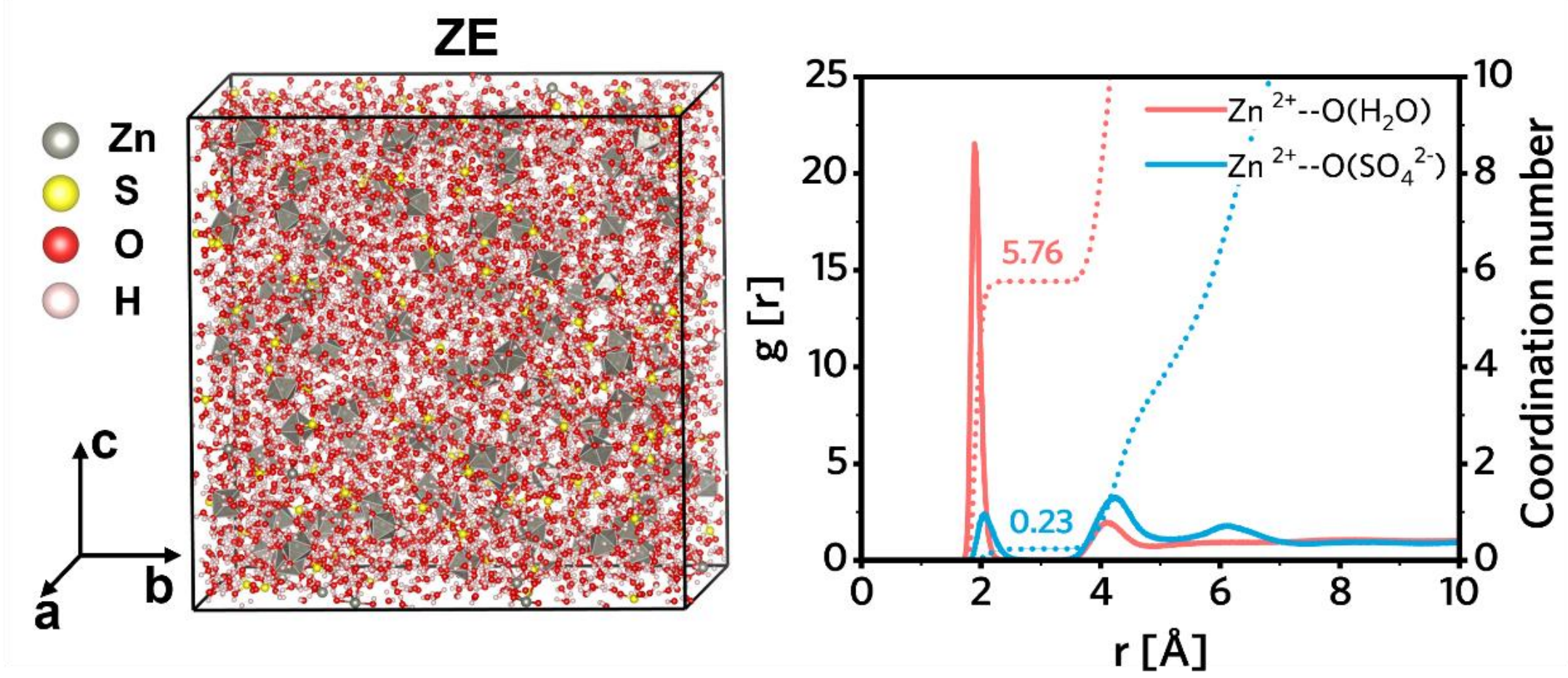

Figure S1. MD snapshot, RDFs, and coordination numbers of Zn^{2+}–$O(H_2O)$ and Zn^{2+}–$O(SO_4^{2-})$ in ZE.

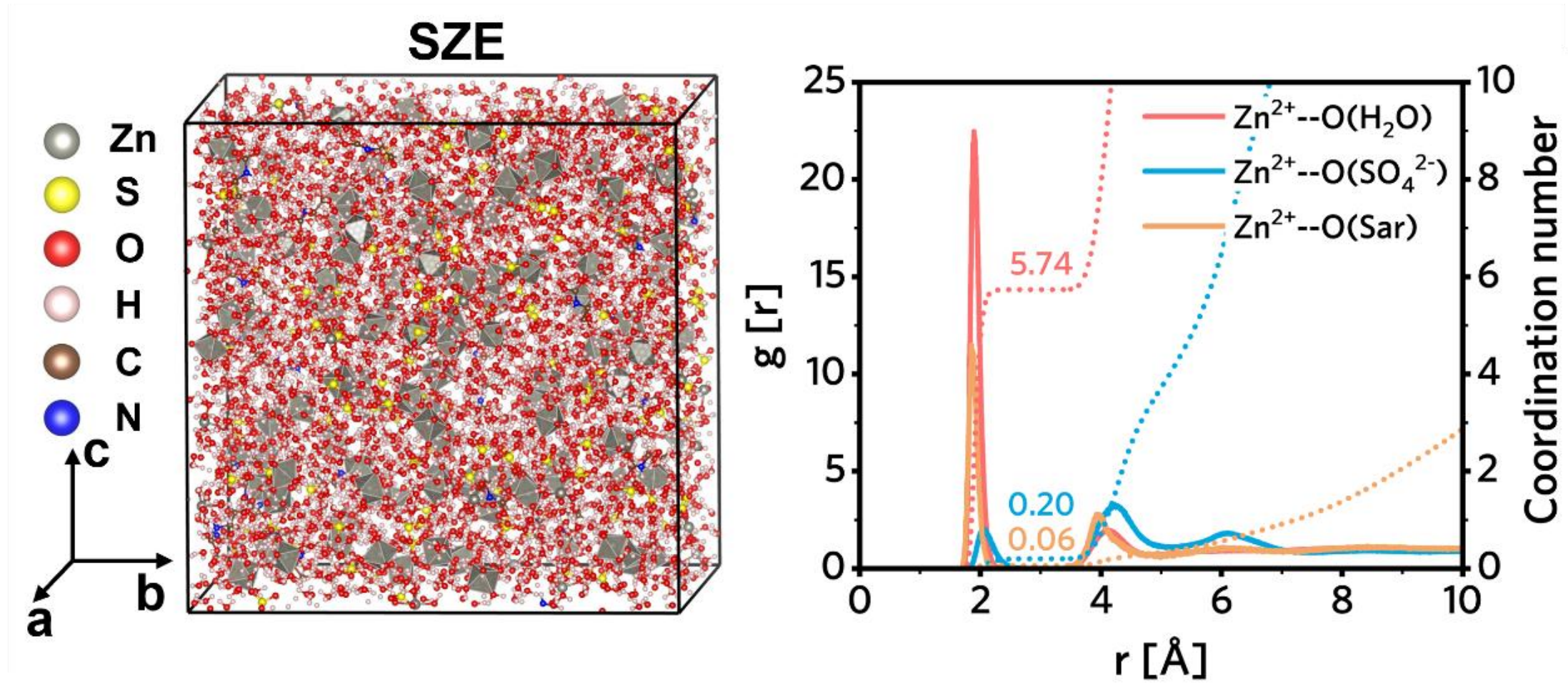

Figure S2. MD snapshot, RDFs, and coordination numbers of Zn^{2+}–$O(H_2O)$, Zn^{2+}–$O(SO_4^{2-})$ and Zn^{2+}–O(Sar) in SZE.

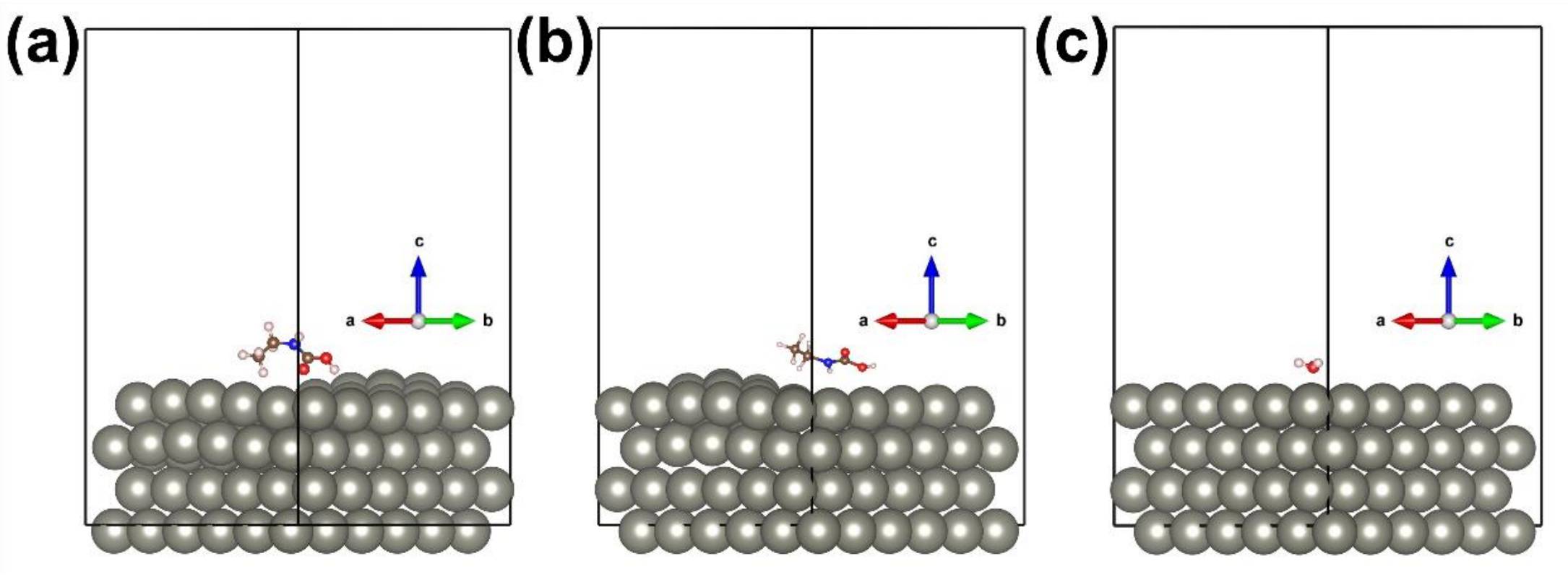

Figure S3. Adsorption models of electrolyte species on the Zn(002) surface. (a, b) Two different adsorption configurations of Sar on Zn(002). (c) Adsorption configuration of H_2O on Zn(002)

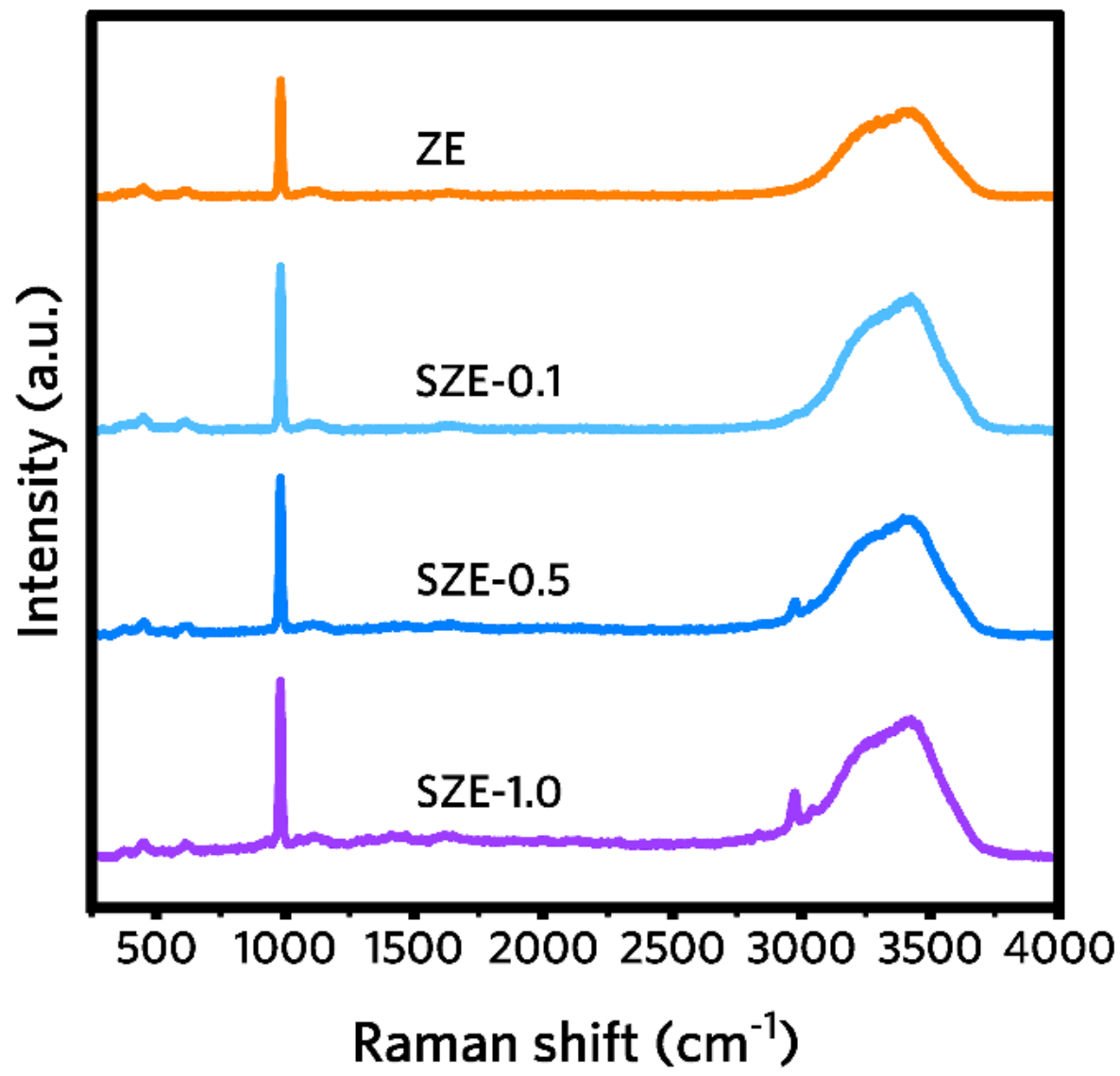

Figure S4. Raman spectra of different hydrogen bond types of the two different electrolyte systems

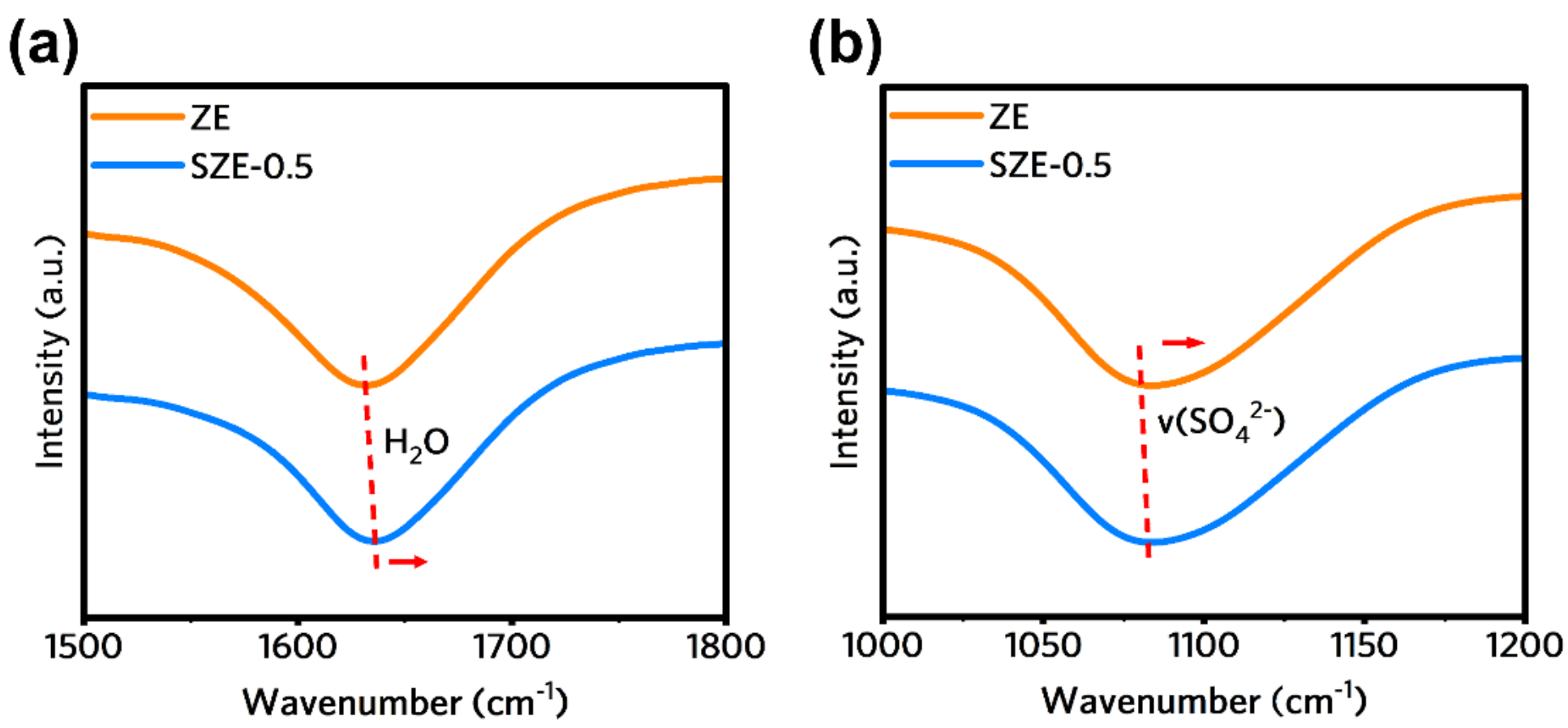

Figure S5. FTIR spectra of ZE and SZE-0.5 showing the characteristic vibrations of (a) H_2O and

(b) v(SO_4^{2-}).

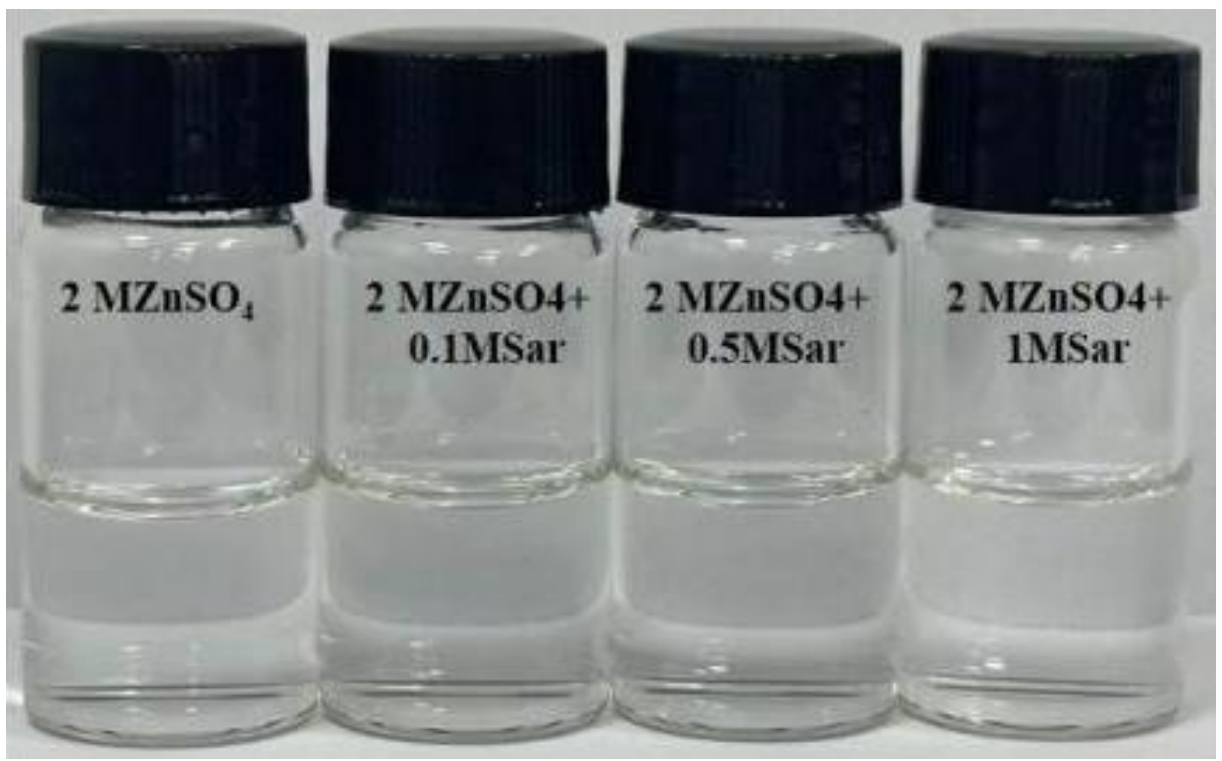

Figure S6. Optical photographs ofdifferent electrolytes after storage at room temperature for one month

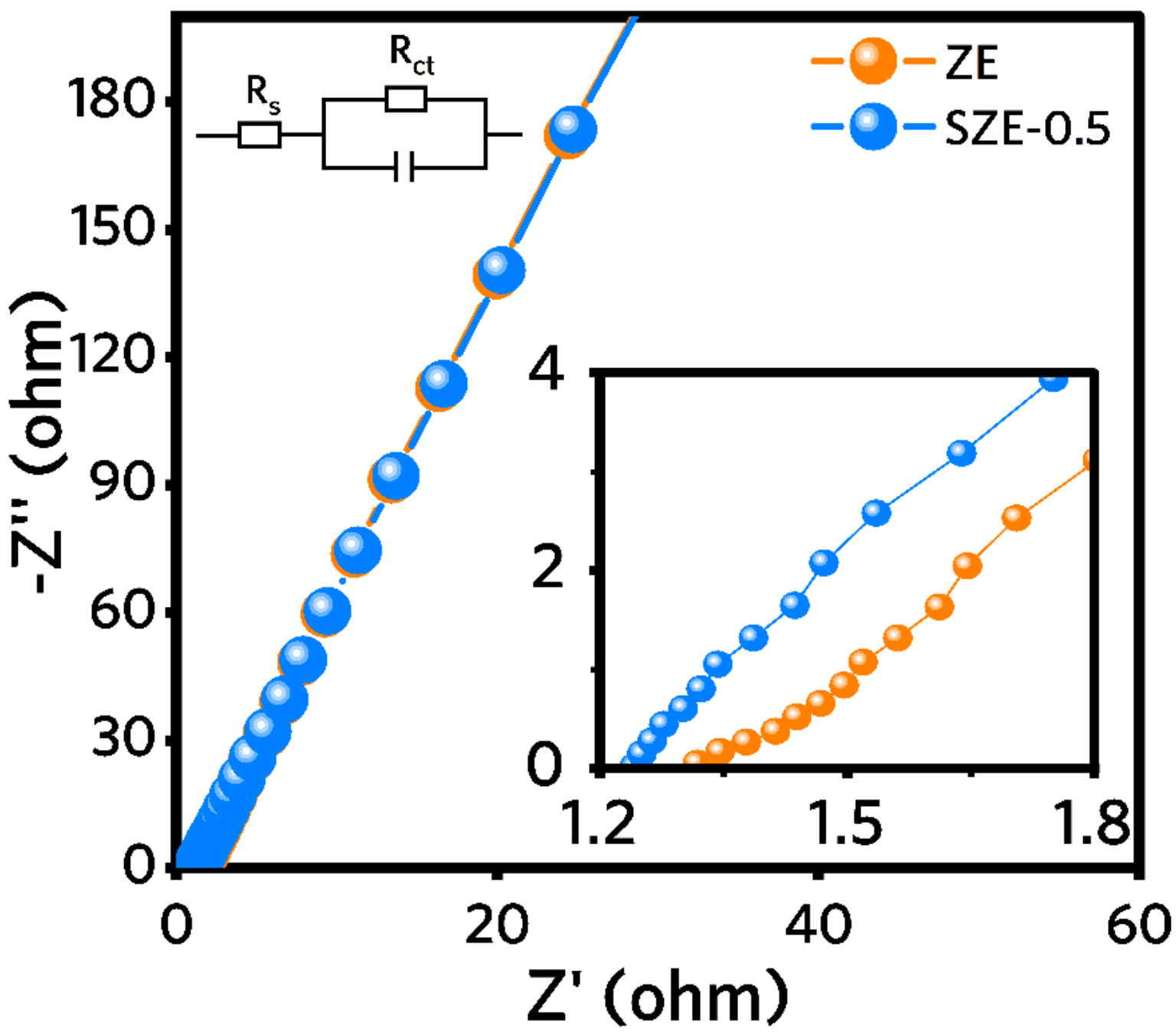

Figure S7. Nyquist plots of ZE and SZE-0.5

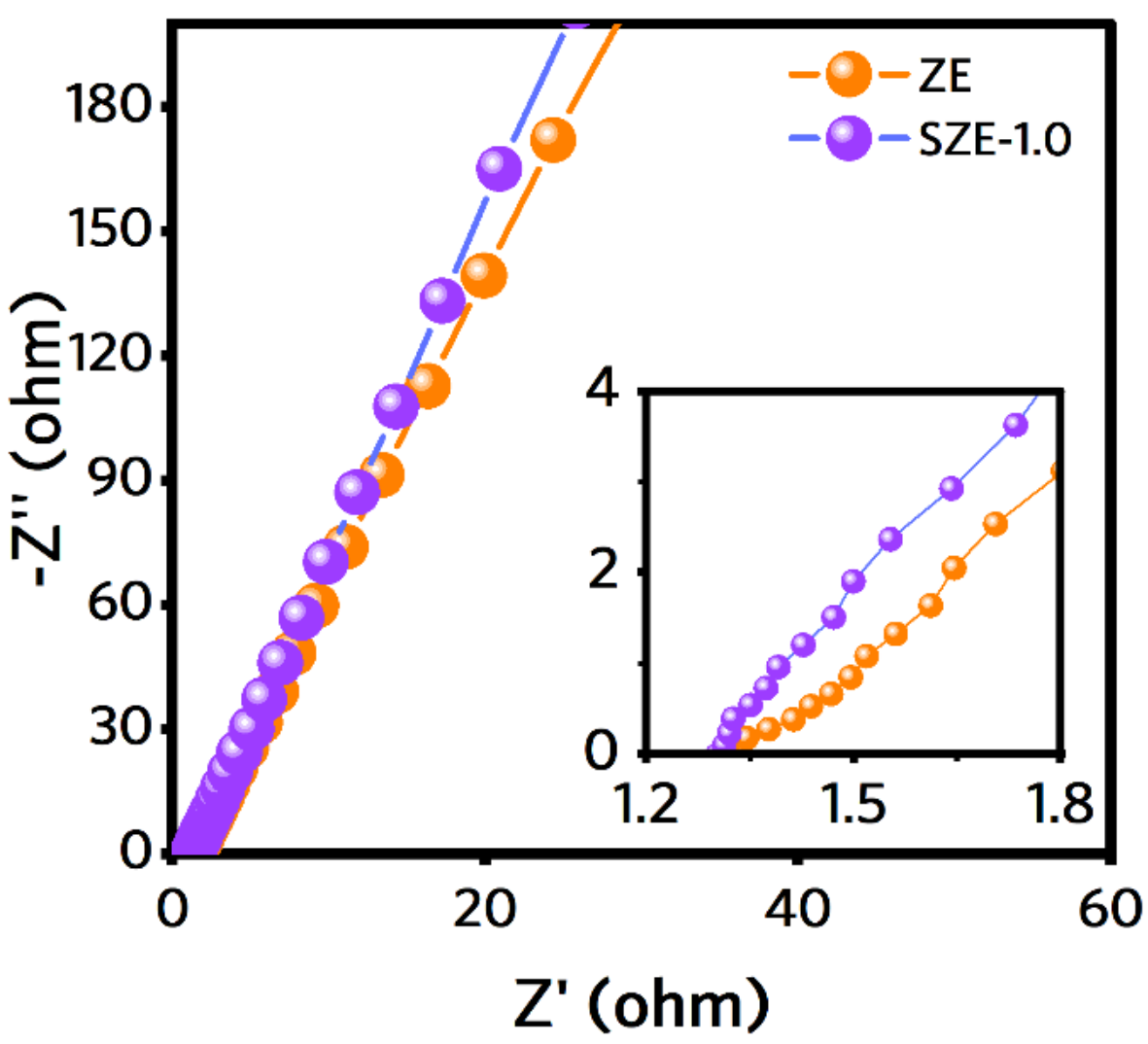

Figure S8. Nyquist plots of ZE and SZE-1.0

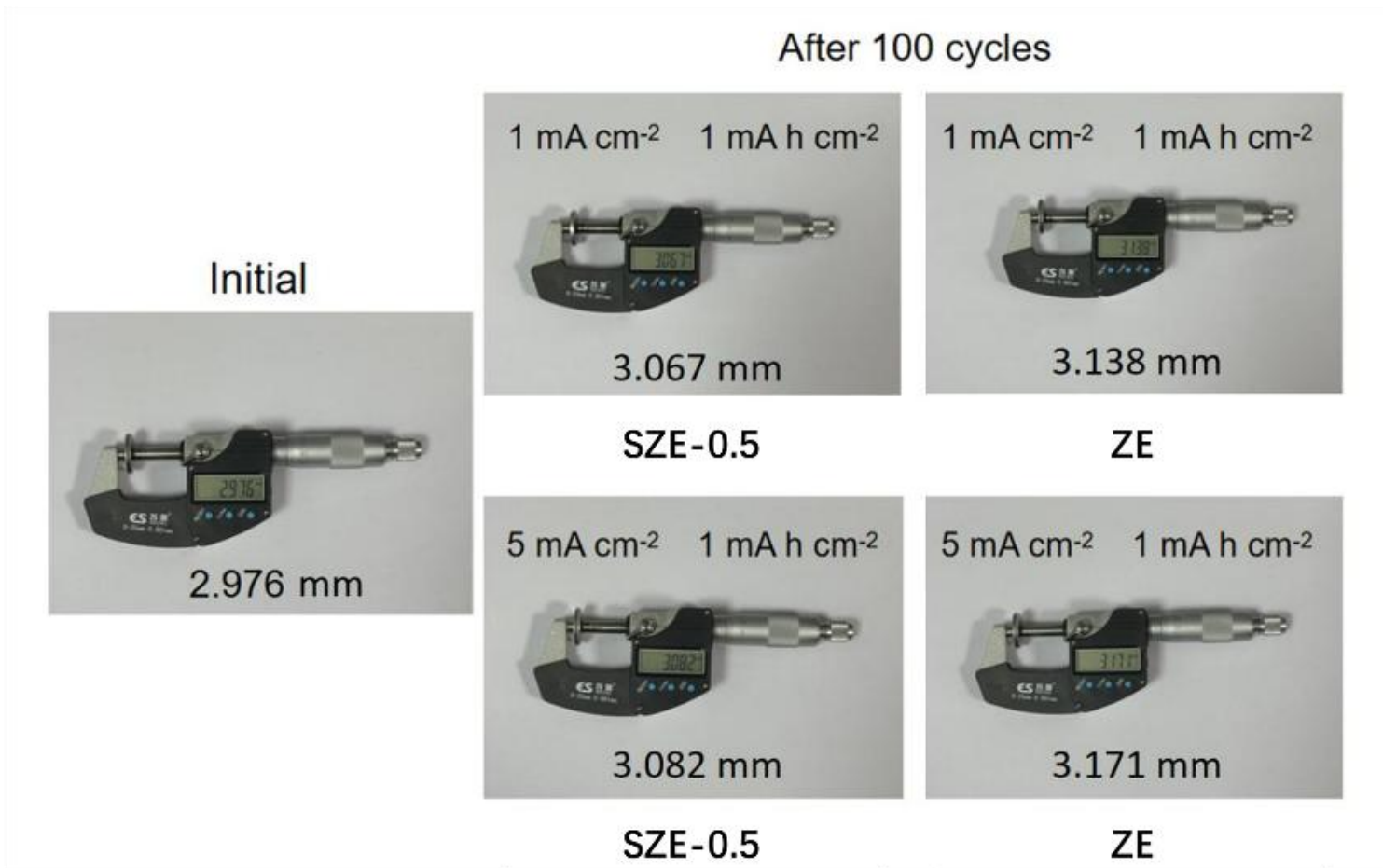

Figure S9. Thickness variation of Zn–Zn symmetric cells assembled with two different electrolyte systems after 100 cycles under different conditions

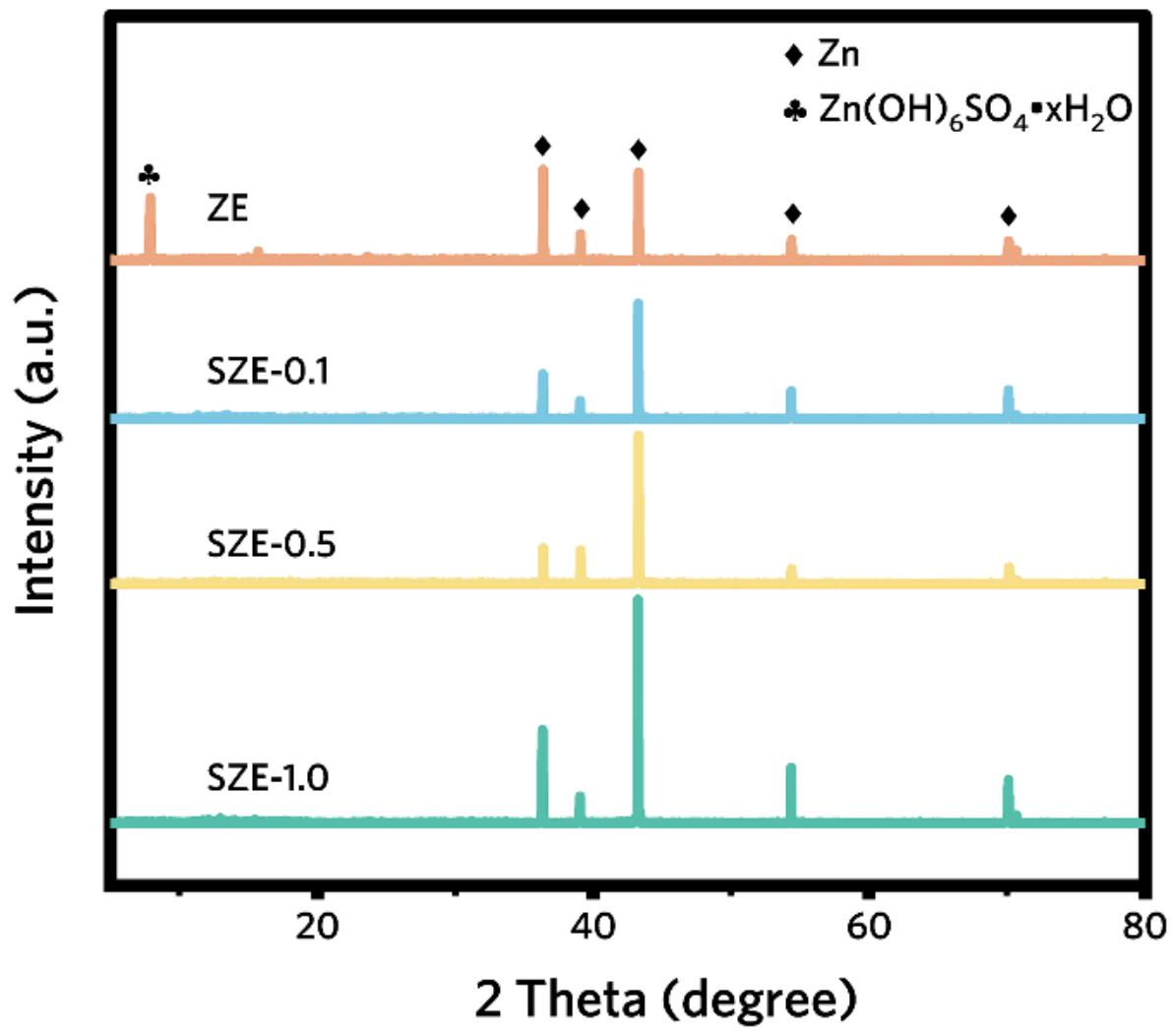

Figure S10. XRD patterns of Zn foils after soaking for 7 days in four different electrolyte systems

Table S1. Charge-transfer resistance (Rct) obtained from fitting the EIS spectra of ZE and SZE-0.5 at different temperatures.

Temperature / ℃	Rct of ZE / Ω	Rct of SZE-0.5 / Ω
25	1034.4	371.4
35	863.7	231.0
45	719.5	216.0
55	626.8	169.7
65	404.8	116.0

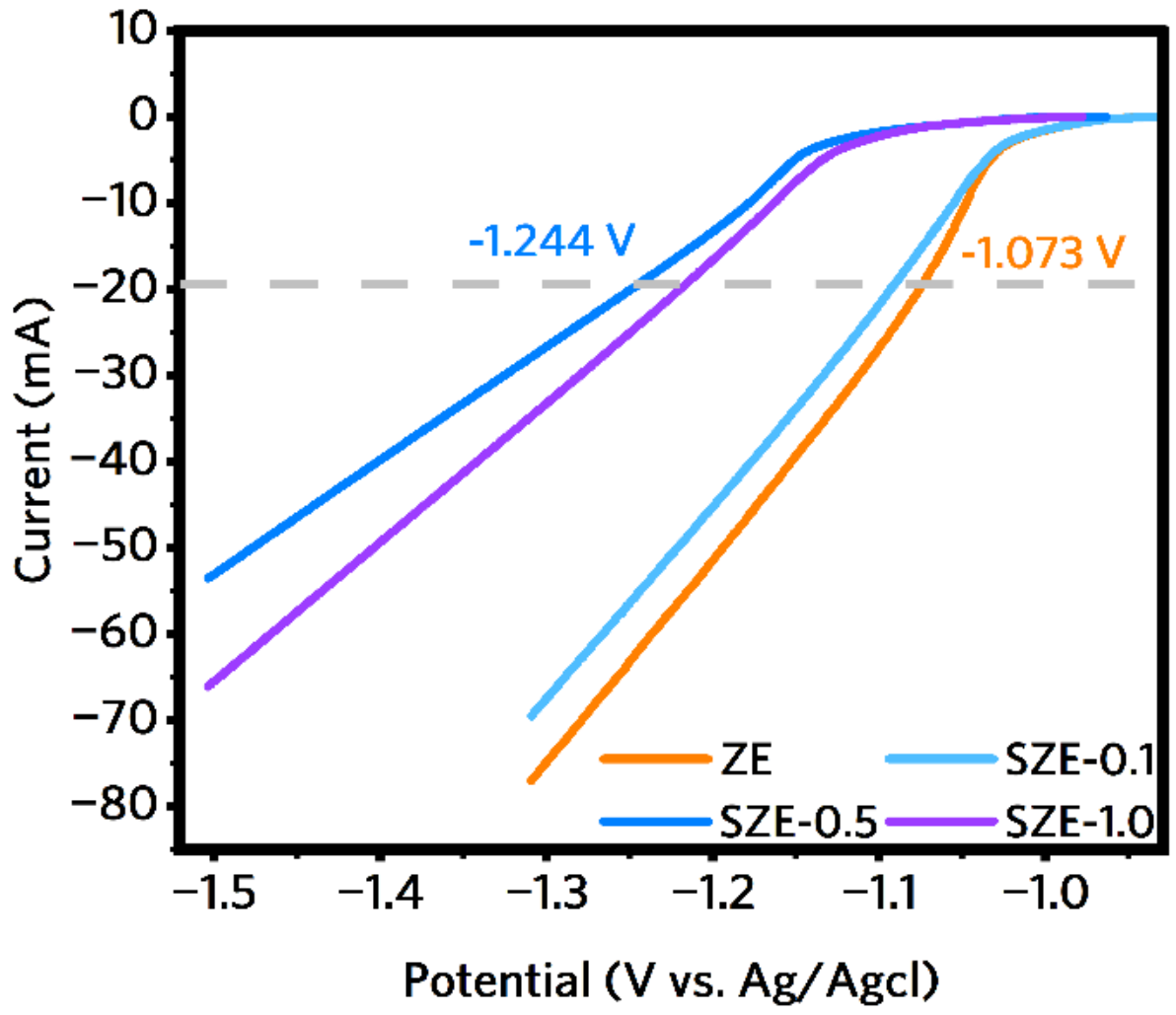

Figure S11. Linear sweep voltammetry (LSV) curves of the four different electrolyte systems

References

Frisch M. J., Trucks G. W., Schlegel H. B., Scuseria G. E., Robb M. A., Cheeseman J. R., et al. (2016). Gaussian 16. Wallingford, CT: Gaussian, inc.

Stephens P. J., Devlin F. J., Chabalowski C. F., and Frisch M. J. (1994). Ab initio calculation of vibrational absorption and circular dichroism spectra using density functional force fields. *J. Phys. Chem.* 98, 11623-11627. doi:10.1021/j100096a001

Krishnan R., Binkley J. S., Seeger R., and Pople J. A. (1980). Self-consistent molecular orbital methods. XX. A basis set for correlated wave functions. *J. Chem. Phys.* 72, 650-654. doi:10.1063/1.438955

Grimme S., Antony J., Ehrlich S., and Krieg H. (2010). A consistent and accurate ab initio parametrization of density functional dispersion correction (DFT-D) for the 94 elements H-Pu. *J. Chem. Phys.* 132, 154104. doi:10.1063/1.3382344

Grimme S., Ehrlich S., and Goerigk L. (2011). Effect of the damping function in dispersion corrected density functional theory. *J. Comput. Chem.* 32, 1456-1465. doi:10.1002/jcc.21759

Lu T., and Chen F. (2012). Multiwfn: A multifunctional wavefunction analyzer. *J. Comput. Chem.* 33, 580-592. doi:10.1002/jcc.22885

Humphrey W., Dalke A., and Schulten K. (1996). VMD: visual molecular dynamics. *J. Mol. Graph* 14, 33-38. doi:10.1016/0263-7855(96)00018-5

Kresse G., and Furthmüller J. (1996). Efficient iterative schemes for ab initio total-energy calculations using a plane-wave basis set. *Phys. Pev. B* 54, 11169. doi:10.1103/PhysRevB.54.11169

Perdew J. P., Burke K., and Ernzerhof M. (1996). Generalized gradient approximation made simple. *Phys. Rev. Lett.* 77, 3865. doi:10.1103/PhysRevLett.77.3865

Momma K., and Izumi F. (2008). VESTA: a three-dimensional visualization system for electronic and structural analysis. *Appl. Crystallogr.* 41, 653-658. doi:10.1107/S0021889808012016

Abraham M. J., Murtola T., Schulz R., Páll S., Smith J. C., Hess B., and Lindahl E. (2015). GROMACS: High performance molecular simulations through multi-level parallelism from laptops to supercomputers. *SoftwareX* 1, 19-25. doi:10.1016/j.softx.2015.06.001

Jorgensen W. L., Maxwell D. S., and Tirado-Rives J. (1996). Development and testing of the OPLS all-atom force field on conformational energetics and properties of organic liquids. *J. Am. Chem. Soc.* 118, 11225-11236. doi:10.1021/ja9621760

Jorgensen W. L., and Tirado-Rives J. (2005). Potential energy functions for atomic-level simulations of water and organic and biomolecular systems. *Proc. Natl. Acad. Sci.* 102, 6665-6670. doi:10.1073/pnas.0408037102

Dodda L. S., Vilseck J. Z., Tirado-Rives J., and Jorgensen W. L. (2017). 1.14* CM1A-LBCC: localized bond-charge corrected CM1A charges for condensed-phase simulations. *J. Phys. Chem. B* 121, 3864-3870. doi:10.1021/acs.jpcb.7b00272

Dodda L. S., Cabeza de Vaca I., Tirado-Rives J., and Jorgensen W. L. (2017).

LigParGen web server: an automatic OPLS-AA parameter generator for organic ligands. *Nucleic Acids Res.* 45, W331-W336. doi:10.1093/nar/gkx312

Darden T., York D., and Pedersen L. (1993). Particle mesh Ewald: An N log (N) method for Ewald sums in large systems. *J. Chem. Phys.* 98, 10089-10092. doi:10.1063/1.464397
